# Factorial validity and measurement invariance of the psychological need satisfaction in exercise scale across gender

**DOI:** 10.1371/journal.pone.0269155

**Published:** 2022-06-07

**Authors:** Abdulwali Sabo, Yee Cheng Kueh, Rabiu Muazu Musa, Frank J. H. Lu, Garry Kuan

**Affiliations:** 1 Biostatistics and Research Methodology Unit, School of Medical Sciences, Universiti Sains Malaysia, Kubang Kerian, Kelantan, Malaysia; 2 Department of Community Medicine, Federal University Dutse, Dutse, Jigawa state, Nigeria; 3 Centre for Fundamental and Continuing Education, Universiti Malaysia Terengganu, Kuala Terengganu, Malaysia; 4 Department of Physical Education, Chinese Culture University, Taipei, Taiwan; 5 Exercise and Sports Science, School of Health Sciences, Universiti Sains Malaysia, Kubang Kerian, Kelantan, Malaysia; University of Texas at Arlington, UNITED STATES

## Abstract

**Background:**

Based on the self-determination theory, the psychological requirements for competence, autonomy, and relatedness boost beneficial exercise behaviour for healthy living. However, there is no valid, reliable Malay version scale to investigate the extent to which these psychological needs are met. The main purpose of this study was to examine the psychometric properties of a Malay version of the Psychological Need Satisfaction in Exercise (PNSE-M) scale. In addition, the purpose of this study was to confirm the measurement and structural invariance of the PNSE-M across gender.

**Methods:**

The study participants included 919 students (male: 49.6%, female: 50.4%), with a mean age of 20.4 years (standard deviation = 1.5). The participants were selected through convenience sampling. The 18-item PNSE-M was used to measure psychological need satisfaction in exercise. The English version of the PNSE was translated into Malay using standard forward-backward translation. Confirmatory factor analysis (CFA) and invariance tests were performed on the three domains of the PNSE-M model. Composite reliability (CR), average variance extracted (AVE), internal consistency based on Cronbach’s alpha, and test-retest reliabilities using intraclass correlation coefficient (ICC) were also computed.

**Results:**

After some model re-specification, the CFA findings based on the hypothesised measurement model of three factors and 18 items indicated acceptable factor structure (CFI = .936, TLI = .923, SRMR = .054, RMSEA = .059). The CR and AVE values were .864–.902 and .573–.617, respectively. Cronbach’s alpha was .891–.908, and the ICC was .980–.985. The findings supported the full measurement and structural invariance of the PNSE-M for both male and female participants. The CFA model matched the data well for both male (CFI = .926, SRMR = .057, RMSEA = .066) and female (CFI = .926, SRMR = .060, RMSEA = .065) participants.

**Conclusion:**

The PNSE-M with three factors and 18 items is considered to be a valid, reliable instrument for university students in Malaysia. It is valid for use to make meaningful comparisons across gender.

## Introduction

Regular physical activity participation has been associated to a lower incidence of a variety of chronic illnesses, including cardiovascular disease, obesity, diabetes, and some cancers [1]. Global estimates show that physical inactivity was responsible for 9% of fatalities in 2008 [2], and the prevalence of physical inactivity has been reported to be higher in the Malaysian population than globally [3]. In 2013, health care systems around the world lost around 53.8 billion international dollars due to insufficient physical activity [4]. Consequently, insufficient physical activity is said to be a significant risk factor in global mortality, causing enormous economic burdens on national health care systems [5].

Self-determination theory (SDT) has been successfully applied as a framework to comprehensively understand how to motivate physical activity and lifestyle modifications [6]. In the view of SDT [7, 8], physical activity can be an intrinsically fulfilling action that offers comfort and personal vitality and can be both internally and externally motivated [9]. Internal motivation arises as physical activity participation gives intrinsic fulfilment and comfort. In contrast, external motivation drives the performance of physical activities in order to receive rewards or recognition or to escape punishment [6].

The basic psychological needs are among the integral components of the SDT proposed by Deci and Ryan [7, 10], as essential for understanding the internalization of motivational development and well-being. According to SDT, the basic psychological needs are separate from any motivational drive or desire assisting behaviour, but instead refer to the innate inclinations desiring fulfilment [7, 11]. The concept of the basic psychological needs as proposed by Deci and Ryan [10] within the SDT has faced some dispute. However, its inclusion within the SDT framework describes an array of motivational phenomena and offers interventional approaches that will promote adaptive behavioural change to intensify the quality of life [12].

SDT has become the most popular framework for exploring motivational issues related to physical activity [13]. Essential components of the SDT framework required for optimal psychological growth and well-being are basic psychological needs, which have a general influence on human motives and enjoyment [7, 14]. Consequently, environments that provide these states of mind improve well-being, whereas settings that limit needs satisfaction hinder motivational growth and promote ill-being [11, 12, 15]. The overall feeling of wellness is associated with individuals’ level of activity and satisfaction of deep psychological needs [6].

According to SDT, satisfying fundamental psychological needs is critical to maintaining and improving individuals’ motivation while engaging in certain behaviours [11]. There are three distinct basic psychological needs considered to be inherent, evident in all developmental stages, and general across cultures: autonomy, competence, and relatedness [11]. Autonomy refers to individuals’ desire to make their own choices with absolute determination. Competence describes the ability to achieve one’s desired outcomes, establish a relationship with his surroundings, and influence one’s actions. Finally, relatedness consists of perceptions of supportive social networks and strong relationships in one’s daily interactions [14]. Fulfilment of these three needs is required for good psychological health [14].

A prior study discovered that greater fulfilment of psychological needs for perceived competence, autonomy, and relatedness was connected with more positive feelings following regular exercise sessions [16]. After controlling for the effects of self-actualization, popularity, safety, and physical flourishing in exercise situations [16], these correlations remained stable. Basic psychological needs satisfaction has also been shown to be a strong predictor of self-efficacy [17]. Similarly, in a study in Malaysia, university students with high levels of self-efficacy and positive thoughts and behaviour were more likely to be physically active despite encountering numerous difficulties [18].

Several studies have examined the positive influence of these three psychological needs on exercise behavior [19–22]. Researchers typically have used either the Basic Psychological Needs in Exercise Scale (BPNES) [23] or the Psychological Need Satisfaction in Exercise Scale (PNSE) [24] to assess psychological need satisfaction related to exercise. The PNSE was originally developed and confirmed to be a valid, reliable instrument among Canadian exercise participants [24], while the BPNES was originally developed and confirmed to be a valid, reliable instrument among Greek exercise participants [23]. These scale were further tested for gender invariance and criterion-related validity based on several variables in agreement with the SDT framework [25]. Furthermore, these scales were translated and applied in different parts of the world including Germany [26], Spain [27], Greek, Spanish, Portuguese and Turkish samples [28], USA [29], Iran [30], Mexico [31], and China [32].

Many studies in Malaysia reported that psychological factors such as motivation for physical activity, relative autonomy index, and enjoyment were among the predictors of the frequency and the extent of participating in leisure-time physical activities among undergraduate students [33, 34]. However, there is yet no available Malay version of the psychological needs satisfaction scale. There is a need to establish a valid measurement scale to evaluate the satisfaction of psychological needs for exercise among university students in line with an earlier study confirming the psychometric properties of the PNSE among university students (mean age = 22.03 years; SD = 4.16) in western Canada [24]. Most university students are in a stage of independence and can make their own lifestyle choices [35]. According to Haerens, Kirk [35], students with higher autonomous motivation are more engaged in physical activity during secondary school and in early adulthood.

Given that various cross-sectional studies have demonstrated the influence of the satisfaction of psychological needs on self-determined motivation [36], education [37, 38], exercise [39], sport [40], and prosocial behavior [41], the purpose of this study was to investigate the psychometric properties of the Malay version of the PNSE (PNSE-M) and to assess its measurement invariance across gender among Malaysian university students.

## Methods

### Participants

The sample comprised 919 undergraduate students (male: *n* = 456, 49.6%; female: *n* = 463, 50.4%), with the mean age of 20.4 years (*SD* = 1.5) studying different courses at the Universiti Sains Malaysia including Medicine, Pharmacy, Nursing, Accounting, Law, Engineering etc. The participants were Malay (81.1%), Chinese (11.3%), Indian (5.0%), and other ethnicity (2.6%), and all were Malaysians with strong comprehension and speaking abilities in Malay language. All the participants reported having at least one session of physical activity per week. The participants’ reported sports activities were basketball, football, badminton, netball, cycling, tennis, and jogging. The mean frequency of weekly exercise participation was 2.8 days (SD = 1.5), with a total mean duration of 64.1 minutes (SD = 41.0). The vast majority of individuals (92.8 percent) were free of disease and were not taking any drugs or medications.

### Measures

The PNSE scale was a self-administrated questionnaire used to assess participants’ psychological need satisfaction during exercise [24]. The PNSE consisted of 18 items and three factors, each with six items. The participants were asked to score their perceptions of competence, autonomy, and relatedness during regular exercise sessions using a six-point rating scale (1 = false, 6 = true) [24, 25]. The internal consistency of the original English version of the PNSE was excellent, with Cronbach’s alpha of 0.90–0.91 across the three factors [24].

### Questionnaire translation

After obtaining permission from the original author, the English version of the PNSE scale was translated into the Malay language. The typical forward-backward translation procedure was used to ensure the quality of the translation, [42]. First, a multilingual speaker translated the PNSE scale from English to Malay. Second, the Malay version was back translated into English by a multilingual Malay speaker. Finally, a panel of five multilingual specialists in health psychology, sport sciences, physical education, and sports psychology assessed the contents of the two translated versions, comparing each item to the original English version. The items were then evaluated further to verify that they were culturally suitable for Malaysians. After the panel achieved an agreement, the final version was sent to a group of 30 undergraduate students for testing. During the pretest, students were asked to remark on the clarity and comprehensibility of the items. Because the students’ input was consistent with one another, no further changes were necessary. The PNSE-M was then tested on a larger sample size for validation study. The PNSE-M is available upon request from the corresponding authors.

### Data collection

The data were collected between September and December 2018 at the Universiti Sains Malaysia using a convenience sampling approach. The study received approval by the Universiti Sains Malaysia’s Human Research Ethics Committee (USM/JEPeM/18070305), and was conducted in accordance with the Declaration of Helsinki. The study employed a cross-sectional design and used the self-reported PNSE-M. Students were contacted at the conclusion of lectures and instructed about the study and data collecting process, and those who agreed to participate in the study were given research information sheets before being requested to complete the questionnaire. When individuals freely completed the PNSE-M questionnaire and returned it to the researchers, they were deemed to have granted implied consent. The PNSE-M was expected to take 10–15 minutes to complete.

Of the 950 questionnaires distributed, 919 questionnaires were returned to the researchers with complete responses to all items. As a result, the final sample consisted of 919 questionnaires (96.7%response rate) with complete replies. 14 days after they filled-in the questionnaire for the first time, 119 participants completed and returned the PNSE-M scale for the second time to analyse its test-retest reliability.

### Statistical analysis

Mplus 8.0 [43] was used to conduct confirmatory factor analysis (CFA) of the initial hypothesized PNSE-M model. The final data obtained in this study were assessed for multivariate normality assumption using the Mardia multivariate skew and kurtosis tests. The results of the multivariate skew (p-value < .001) and kurtosis (p-value < .001) tests indicated a violation of normality assumption. Consequently, the robust maximum likelihood estimator (MLR) was the preferred CFA method because it was robust to non-normal data distributions and provided robust parameter estimates [43]. To demonstrate appropriate psychometric qualities of the PNSE-M, a factor loading of .40 [44] or above was employed as a cut-off value. Items with factor loadings of less than .40 were considered doubtful and were eliminated only if there was adequate theoretical evidence.

To fit a model with a sample size of more than 250 and observed variables of more than 12 but less than 30, the following recommended fit indices and cut-off point values were used: root mean square error of approximation (RMSEA) of less than .07, standardised root mean square residual (SRMR) of less than .08, and comparative fit index (CFI) or Tucker and Lewis index (TLI) of .92 or more [45]. The construct reliability of the PNSE-M constructs was assessed using composite reliability (CR) and average variance extracted (AVE). CR was computed using Raykov’s method [46] in Mplus 8.0. The recommended values were greater than or equal to .60 for CR [47] and .50 for AVE [48]. Discriminant validity was established when the correlation between factors was less than .85 [49], and the AVE value of each factor was higher than its squared correlation with other factors (i.e., the shared amount of variance) [48]. Model re-specification was also carried out by adding the residual covariances between items within the same factor. The model re-specification was carried out when the researchers had appropriate theoretical justification and was based on the modification index (MI) values.

Based on the suggested criteria for establishing model measurement invariance [43, 50, 51], a hierarchical test of measurement invariance was performed across gender. The model parameters were subjected to sequential stringent restrictions, and changes in the model fit indices were assessed. To verify measurement invariance, the configured invariance model had to be established and compared to the fit indices for other invariance models. The configural invariance model placed no gender equality constraints on model parameters. The weak invariance or metric invariance model was then constructed and tested. The weak invariance model placed equality constraints on model factor loadings across gender and ensured the measuring scale’s similarity across gender to provide valid comparisons. Third, the strong invariance model was developed and tested. The strong invariance model placed equality constraints on the factor loadings and item intercepts across gender, allowing the scale factors to be compared across gender. Finally, a strict invariance model was developed and tested. To ensure that the error item variances were invariant across gender, the rigorous invariance model put equality constraints on the factor loadings, item intercepts, and residual variances.

Following the evaluation of measurement invariance, the structural invariance of the model parameters was investigated. The factor variance and covariance invariance were calculated to see if the associations between the factors remained consistent across gender. The factor means invariance was then investigated to determine the factor mean differences between genders. The structural invariance measured the amount of difference between the male and female samples and had no relation to the measurement scale investigated. The suggested cut-off values for the measurement and structural invariances from a less restrictive model to a more restrictive model were an absolute difference (Δ) of .01 or less for CFI (ΔCFI) and TLI (ΔTLI) and .015 for RMSEA (ΔRMSEA) [50, 52, 53].

In this study, we also assessed the internal consistency of the PNSE-M using Cronbach’s alpha and compared it to the Cronbach’s alpha of previous studies [24, 54, 55]. The intraclass correlation coefficient (ICC) was calculated based on test-retest reliability with 14 days interval between the first measurement and second measurement using a subsample of 119 individuals’ scores. Greater than .90 ICC values were considered to indicate excellent stability [56]. SPSS 26.0 was used to compute Cronbach’s alpha and ICC.

## Results

### Factorial validity of the PNSE-M

**Fig 1:**
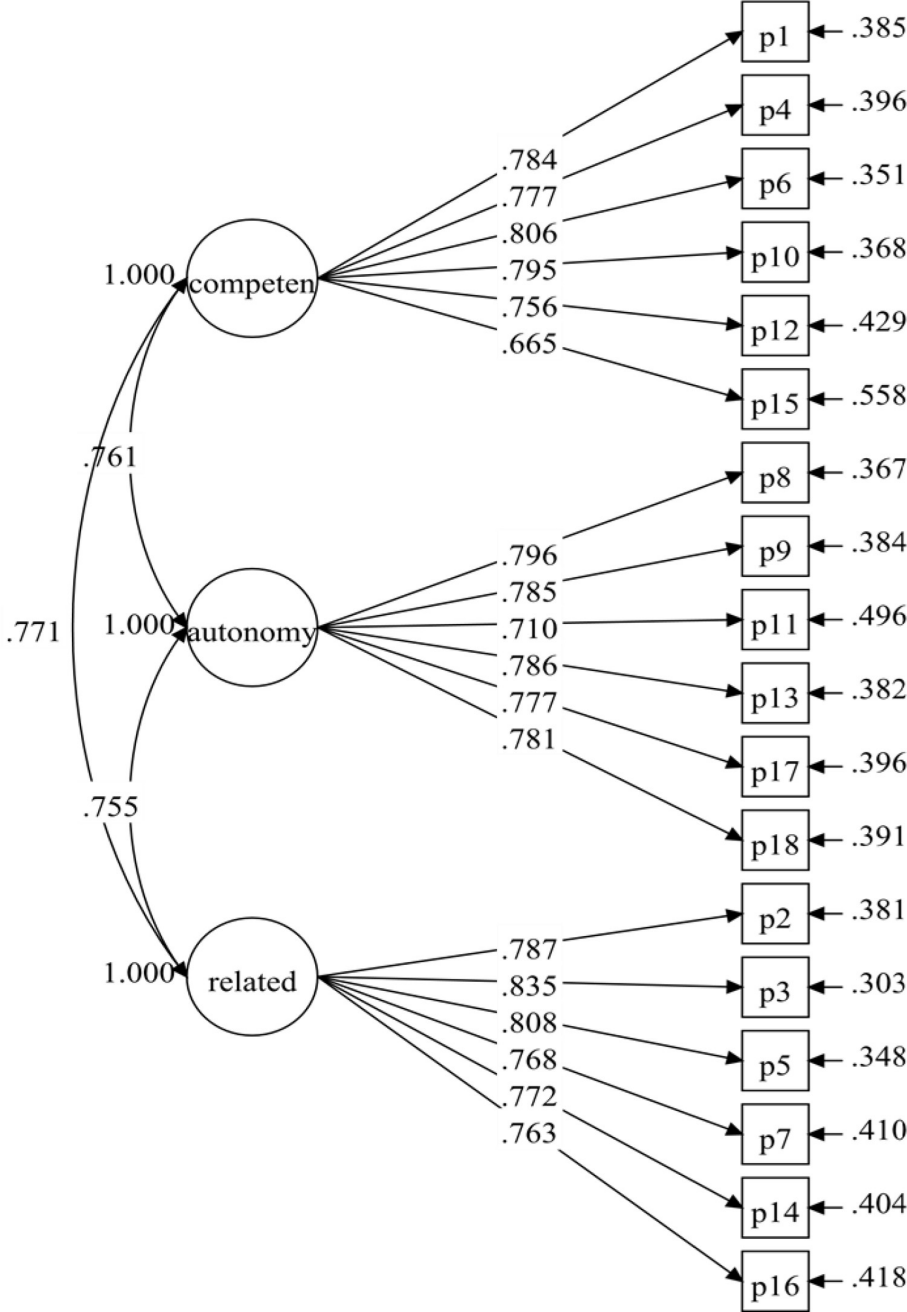
PNSE-M model (model-1).

**Fig 2:**
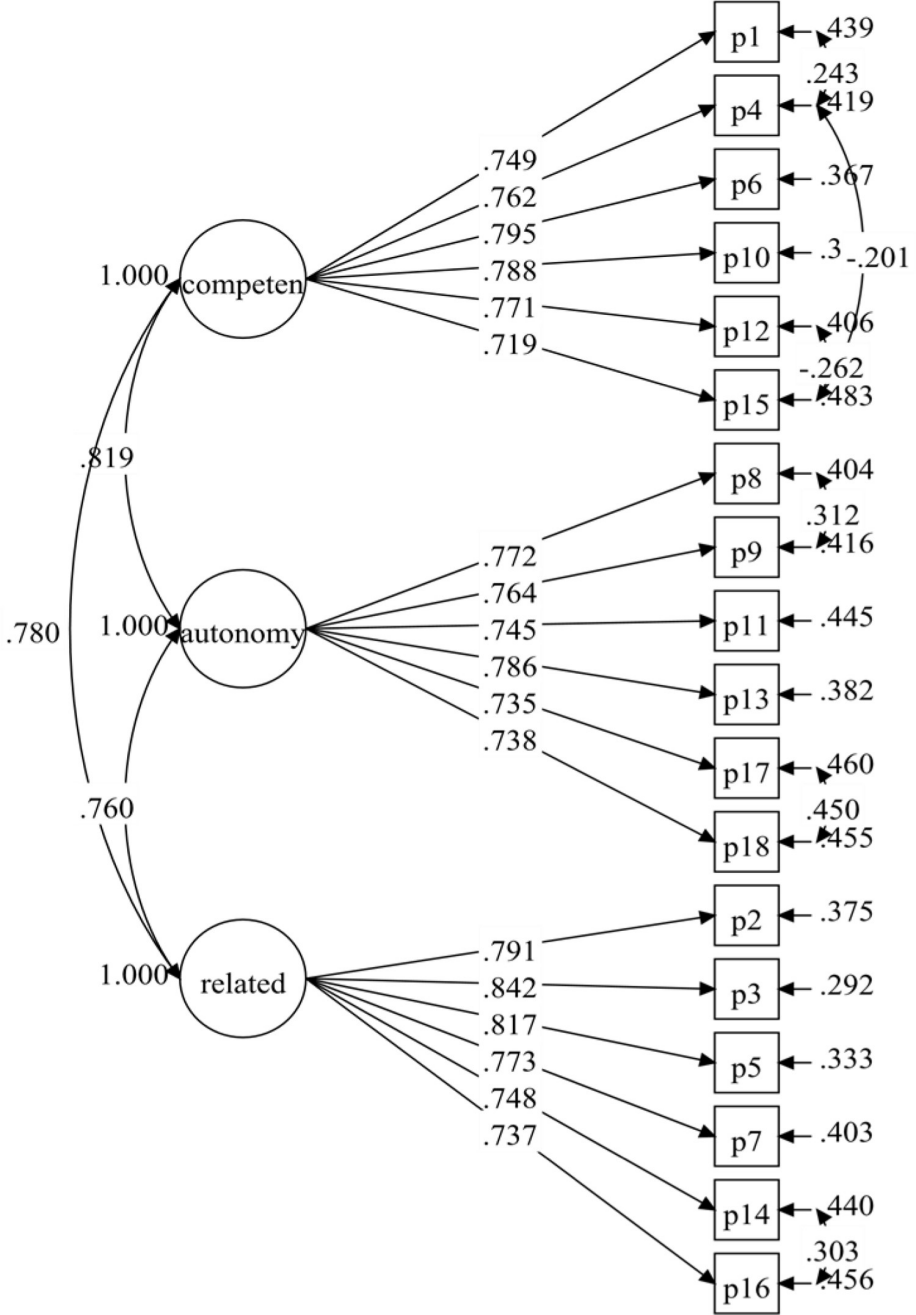
PNSE-M model (model-2).

The PNSE-M proposed measuring model had 18 items and three correlated latent variables (six items each). The first model examined (Model 1) did not produce adequate fit indices (Table 1). However, with a *p*-value < .001, all of the standardised factor loadings were larger than .60 (Fig 1). Model 1 was improved by correlating the item residuals within the same factor based on the modification indices and after discussion among researchers (see Table 1). The findings (Model 2) demonstrated an excellent match to the data (see Table 1). Model 2 was thus verified, with the necessary fit indices and standardised factor loadings of .719–.842, and all items retained (Fig 2).

**Table 1:** Summary for PNSE-M model fit indices.

Path model	RMSEA (90% CI)	CFI	TLI	SRMR
Model-1	.073 (.068, .078)	.900	.884	.063
Model-2^a^	.059 (.054, .065)	.936	.923	.054

^a^Model-2 with correlated items residual; P18 with P17, P16 with P14, P9 with P8, P1 with P4, P15 with P12, P15 with P4.

### Discriminant and convergent validity

The final PNSE-M model (Model 2) had CR values ranging from .864 to .902, with AVE values ranging from .573 to .617. All of the correlations between the variables were smaller than the required threshold of .85 for discriminant validity. Furthermore, the squared values of these correlations were always less than the components’ AVE values, showing appropriate discriminant validity. Table 2 displays the final PNSE-M model’s CR and AVE values, correlation coefficients, and correlation coefficient squared.

**Table 2:** Composite reliability (CR), average variance extraction (AVE), factor correlation and squared correlation for PNSE-M final model.

Variables	CR (95% CI)	AVE	1	2	3	*r* ^*2*^
1. Competence	.902 (.885, .918)	.584	1	.590**		.348
2. Autonomy	.864 (.843, .885)	.573		1	.577**	.333
3. Relatedness	.896 (.882, .911)	.617	.616**		1	.379

**Correlation is significant at the 0.001 level (two tailed), *r*^*2*^ = squared correlation coefficient.

### Measurement model of the PNSE-M for males and females

**Fig 3:**
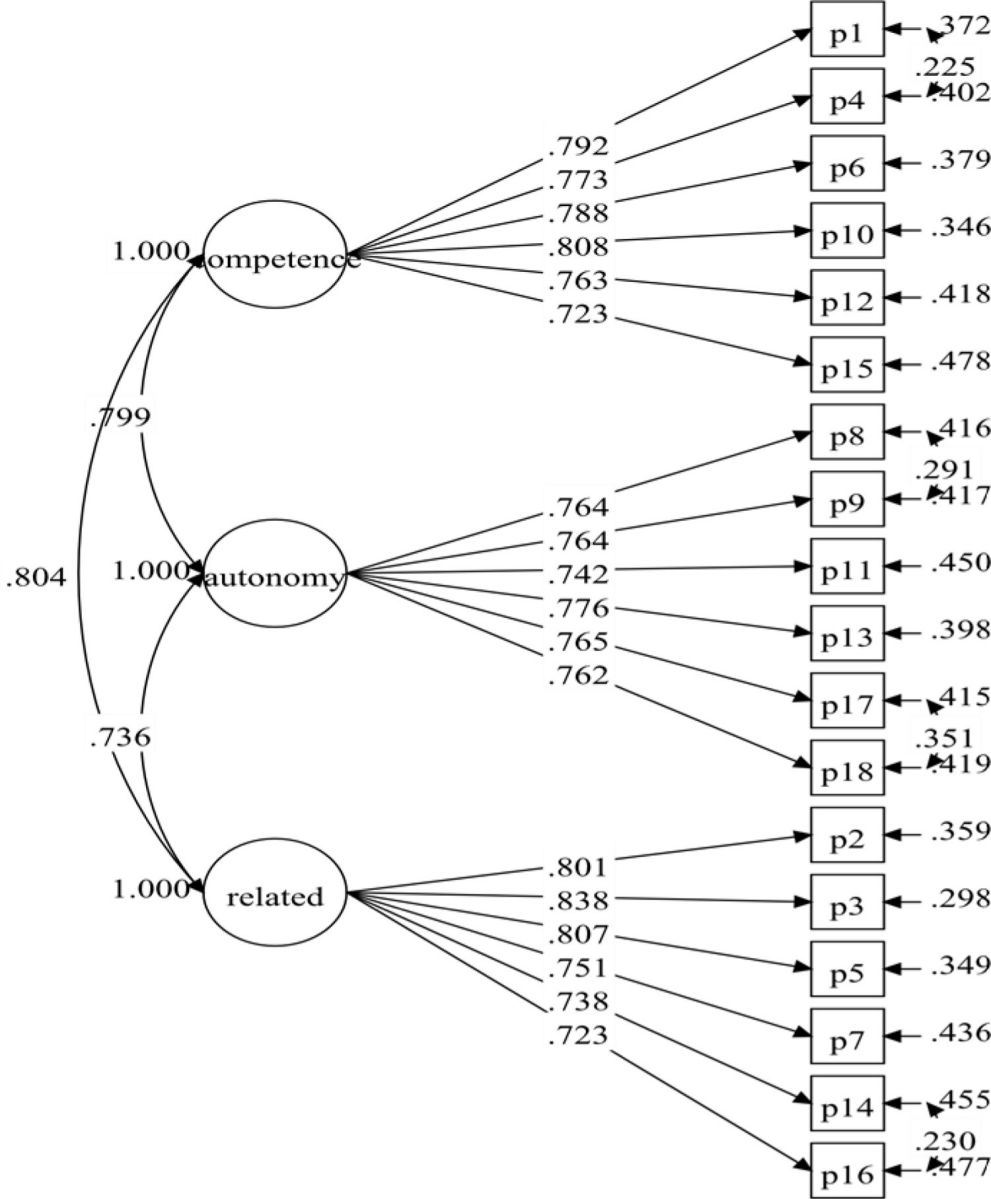
Male PNSE-M model (model-4).

**Fig 4:**
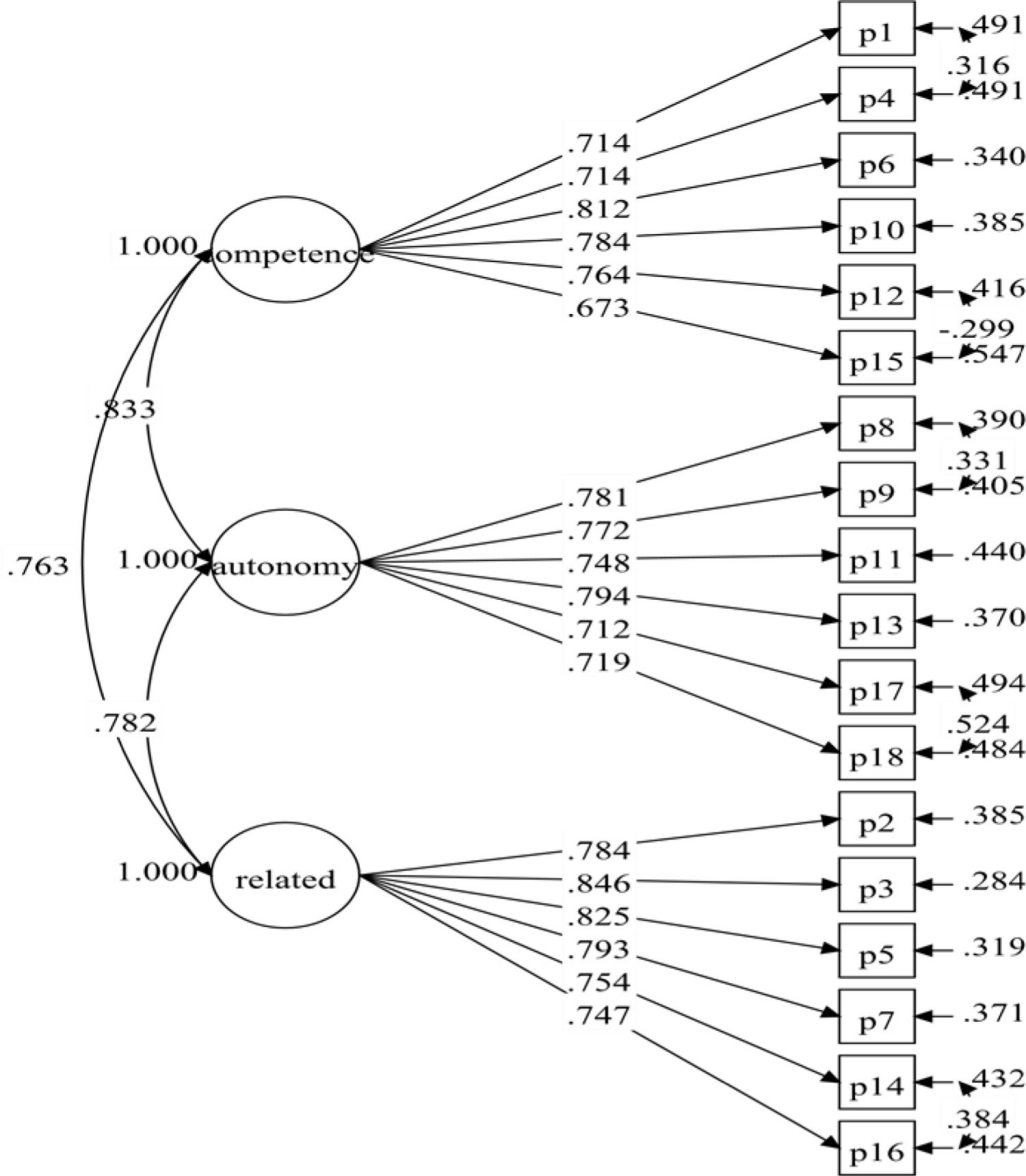
Female PNSE-M model (model-6).

Following the establishment of the overall measurement model based on all of the data, the baseline measurement models for both males (Model 3) and females (Model 5) were determined. These two models did not provide a satisfactory match to the data (Table 3). The fit indices were good after re-specification for both the male model (RMSEA = .066, CFI = .926, TLI = .911, SRMR = .057) and the female model (RMSEA = .065, CFI = .926, TLI = .911, SRMR = .060). The CFI and TLI values of the two models were similar, while the RMSEA and SRMR values were slightly different. The final male model had four additional residual covariances (P18 with P17, P9 with P8, P16 with P14, and P4 with P1), while the final female model had five added residual covariances (P18 with P17, P16 with P14, P9 with P8, P4 with P1, and P15 with P12). The resulting male and female models’ standardised factor loadings were .723 to .838 (Fig 3) and .712 to .846 (Fig 4), respectively.

**Table 3:** The Psychological Need Satisfaction in Exercise Malay version (PNSE-M) baseline model fit results and tests of measurement invariance.

Models	CFI	TLI	RMSEA	SRMR	Model comparison	ΔMLR *X*^2^ (df)^a^, p-value	ΔCFI	ΔTLI	ΔRMSEA
Model-3 (male group: hypothesised)	.908	.893	.072	.061	-	-	-	-	-
Model-4^b^ (male group: re-specified)	.926	.911	.066	.057	-	-	-	-	-
Model-5 (female group: hypothesised)	.879	.860	.081	.070	-	-	-	-	-
Model-6^c^ (female group: re-specified)	.926	.911	.065	.060	-	-	-	-	-
Model-7 (configural)	.929	.914	.064	.057	-	-	-	-	-
**Measurement invariance**									
Mode-8 (weak)	.927	.916	.063	.061	8 versus 7	18.040 (15), .261	-.002	.002	-.001
Model-9 (strong)	.924	.918	.063	.063	9 versus 8	29.141 (15), .015	-.003	.002	0
Model-10 (strict)	.925	.924	.061	.066	10 versus 9	16.748 (18), .540	.001	.006	-.002
**Structural invariance**									
Model-11 (factor variance and covariance)	.925	.920	.062	.065	11 versus 9	4.474 (6), .613	.001	.002	-.001
Model-12 (factor variance, covariance and factor mean)	.924	.921	.062	.066	12 versus 11	4.989 (3). .173	-.001	.001	0

^a^scaled difference in X^2^ for model comparison.

^b^Adding residual covariances between item P18 with P17, P9 with P8, P16 with P14, and P4 with P1.

^c^ Adding residual covariances between P18 with P17, P16 with P14, P9 with P8, P4 with P1, and P15 with P12. Model-1 and Model-2 are reported in Table 1.

### Measurement and structural invariance

All of the items were kept in the baseline models for male and female participants. The configural model invariance was then constructed by integrating the two baseline models with the same number of fixed and free factor loadings. The configural invariance model suited the data well, with no equality constraints put on any parameters across gender (Table 3).

The weak measurement invariance model suited the data well (Table 3) and was compared to the non-restrictive model (the configural model), with these differences indicating satisfactory metric invariance scores across gender (ΔCFI = -.002, ΔTLI = .002, ΔRMSEA = -.001). The findings revealed that the items had similar meanings in both the male and female. Following that, the strong invariance model, which was the second most restrictive model (equality constraints put on factor loadings and item intercepts), demonstrated adequate invariance (ΔCFI = -.003, ΔTLI = .002, ΔRMSEA = 0). The findings revealed that the factor loadings and intercepts were constant across gender. Finally, the rigorous invariance model was proven to have adequate invariance by imposing equality constraints on the factor loadings, item intercepts, and residual variances (ΔCFI = .001, ΔTLI = .006, ΔRMSEA = -.002). The findings suggested that the average item scores were comparable in both the male and female.

The structural invariance of the PNSE-M across gender was also investigated. This structural invariance model fit the data well (CFI = .925, TLI = .920, SRMR = .065, RMSEA = .062), and its differences with the less restrictive invariance model (strong invariance) were within the suggested values (ΔCFI = .001, ΔTLI = .002, ΔRMSEA = -.001). In addition, we evaluated the mean invariance model (Model 12) shown in Table 3 to determine if the factor means were invariant across gender. This model showed good fit indices (CFI = .924, TLI = .921, SRMR = .066, RMSEA = .062), and the differences with the less restrictive model (factor variance and covariance) were within the acceptable values (ΔCFI = -.001, ΔTLI = .001, ΔRMSEA = 0). The findings suggested that the relationships between the three variables of the PNSE-M were consistent across gender, and there was no significant mean difference in the PNSE-M score between the male and female samples.

### Internal consistency

Cronbach’s alpha values were .891, .898, and .908 for competence, autonomy, and relatedness, respectively. The item–total correlation was .572 to .789. When the item-total correlation was greater than .30, the scale was considered to have sufficient internal consistency, and each item contributed sufficiently to the assessment of its factor [57].

### Test-retest reliability

In order to assess the test-retest reliability, 119 participants volunteered to complete the PNSE-M again on day 14. The mean competence scores decreased from 4.19 (SD = .90) to 4.14 (SD = .93), with an ICC value of .982 (95% CI, .974, .987, *p*-value < .001). The mean autonomy scores decreased from 4.84 (SD = .75) to 4.73 (SD = .83), with an ICC value of .985 (95% CI, .978, .989, *p*-value < .001). The mean relatedness scores decreased from 4.57 (SD = .74) to 4.41 (SD = .81), with an ICC value of .980 (95% CI, .972, .986, *p*-value < .001). These ICC values showed that the PNSE-M was stable over two time points.

## Discussion

In the field of exercise and sports psychology, the scientific inquiry needs the establishment of cross-cultural analysis to avoid propagation of theories that cannot be generalized [58]. This is because ethnicity and culture play an important role in explaining the variability of cognition and behaviours in psychological theories of exercise and sports [58]. The SDT has been studied often concerning its cross-cultural applicability. It has been widely reported that the psychological needs for autonomy, competence, and relatedness, are universal in their meaning and their influence [59]. This indicates that need satisfaction improves psychological health, irrespective of cultural setting [59]. However, other cultural-relativists [60] stated that these needs are studied within cultures and that autonomy, for example, portrays a western ideal learned in western cultures that stress individualism instead of socialism and interdependence. This reveals that in eastern cultures, autonomy plays a less important role in people’s wellbeing [61]. According to the SDT, all humans possess some psychological needs that must be satisfied to experience sufficient well-being irrespective of culture [59]. Hence, the present study translated the English version of the PNSE into Malay (PNSE-M) and then test the factor structure and composition of the PNSE-M among university students within the SDT framework [7, 11] using CFA.

The findings supported the psychometric properties of the PNSE-M scale, which was consistent with prior studies [24, 25, 54]. The PNSE-M scale fit the data well, and the findings offered strong evidence for construct validity, measurement and structural invariance across gender. A previous study involved in the initial development of the PNSE tested its equivalence across gender with the aim to construct a single measure that can be interpreted equally and used to compare male and female groups [24]. According to Weinberg, Tenenbaum [62], male and female participants possess distinct underlying motives for physical activity behavior. Females have motives more related to appearance, whereas males have motives more related to competition and ego [62–64]. The PNSE is a psychological measure that relies on the respondents’ subjective judgements, and those with different genders might have different interpretations of the PSNE constructs. As such, proving the invariance of the PNSE-M across gender is important to make meaningful comparisons across gender in Malay population.

In Malaysia, gender was associated with physical activity involvement, with females being less likely to engage than males [65]. Hence, this study presents the Malay version of the PNSE scale that will not only assess the population’s level of psychological need for exercise but also make valid comparisons between the male and female population. The PNSE-M has three factors (i.e. autonomy, competence, and relatedness) with six items in each factor. The items were designed to examine the extent to which participation in exercise behaviour elevates the feelings of autonomy, competence, and relatedness [24]. Each item consisted of 6-point rating options ranging from 1 (False) to 6 (True). Higher scores denote a higher level of psychological need satisfaction, whereas, lower scores denote a lower level of psychological need satisfaction for exercise [24, 25].

All 18 items with strong factor loadings on their corresponding factors were retained in the final PNSE-M model. The construct reliability of the PNSE-M, or the extent to which the items reflected their respective factors, surpassed the required values of .60 for CR [47] and .50 for AVE [48]. These findings suggested that the PNSE-M has adequate construct validity, and all of the items precisely measure their respective factors. The discriminant validity of the scale was evaluated by investigating the factor correlations and comparing them to the AVE values. All the correlations were less than the recommended value of .85 [49] and their corresponding AVE values [48]. These findings suggest adequate discriminant validity, with each factor explaining some information that is different from the other factors.

The needs for autonomy, competence, and relatedness are associated with positive exercise behavior and mental-health-related quality of life [11]. It, therefore, is imperative to establish an instrument that reliably indicates the degree to which these needs are satisfied in exercise contexts. The PNSE-M model tested in this study show adequate internal consistency. Cronbach’s alpha values of .891–.908 appear to be consistent with the results of previous studies (.91–.94 [54], .90–.91 [24], and .92–.93 [25]). The ICC values for test-retest reliability of .980–.985 obtained from this study showed that the PNSE-M scale has excellent reliability [56]. Previous studies related to the need satisfaction constructs reported ICC values of .91–.96 [66] and .97 for all the subscales [23]. These results indicate that the PNSE-M tested in the present study maintain the same number of factors and items as the original translated PNSE [24]. These results were also consistent with other studies related to PNSE scale [26–28, 30, 54, 55]. Other studies tested the BPNSE with three factors and 12 items (i.e. 4 items in each factor) [23, 32]. However, the PNSE-M was tested based on the PNSE with three factors and 18 items.

Additionally, the PNSE-M’s measurement and structural invariance across gender were satisfied [50, 67]. These findings imply that the male and female samples had equivalent understandings of all the 18 items in the PNSE-M. This equivalent of understanding is required in order to perform accurate comparisons of male and female students’ satisfaction with exercise needs. The structural invariance of the PNSE-M was favorable for factor variance and covariance, and the factor means were invariant across gender. These findings suggested that the magnitude of relationships between the factors remains consistent across male and female groups, and there was no significant mean difference of total PNSE-M score between the males and females. These findings were also consisted with the original PNSE scale that confirmed than the PNSE can be applied to make comparisons between male and female perception of psychological needs satisfaction for exercise [24].

In the present study, the model fit indices were improved by adding error covariances between items of the same factor. These error covariances represent shared sources of variability over and beyond the factors in the model [47].The error covariances added in this study for the final overall model, male model, and female model were 6, 4 and 5 respectively. The error covariances between items for items P18 (I feel like I am the one who decides what exercises I do) with P17 (I feel free to choose which exercises I participate in), P16 (I feel like I get along well with other people who I interact with while we exercise together) with P14 (I feel connected to the people who I interact with while we exercise together), P9 (I feel free to make my own exercise program decisions) with P8 (I feel free to exercise in my own way) and P1 (I feel that I am able to complete exercises that are personally challenging) with P4 (I feel confident I can do even the most challenging exercises) were added for both the final overall model, male model, and female model. The error covariance for items P15 (I feel good about the way I am able to complete challenging exercises) with P12 (I feel like I am capable of doing even the most challenging exercises) is added for the final overall model and the female model. The error covariance for items P15 (I feel good about the way I am able to complete challenging exercises) with P4 (I feel confident I can do even the most challenging exercises) was added for the final overall model. These covariances were based on MI values reported in Mplus and with adequate theoretical support. Previous studies have reported that error covariances can be added when they make a theoretical meaning [68, 69].

There are several limitations to this study. First, the results should be interpreted with caution because the data were collected from a single university. Nonetheless, the large study sample size strengthens the findings’ generalisability. Second, self-administered questionnaire has been linked to response bias. This bias has the potential to reduce the accuracy of the data obtained for the study. In order to reduce this bias, the questions were answered anonymously, and participants were asked to be honest in their responses. Lastly, the present study does not include other validity testing, such as concurrent and predictive validity. As a result, future study should consider incorporating other validity testing to strengthen the quality of the PNSE-M.

This study indicated that the PNSE-M scale may be efficiently administered as a valid, reliable tool to measure university students’ psychological needs for competence, autonomy, and relatedness in exercise. Future study should examine the replicability of the PNSE-M with a more diversified Malay population with different ages, education levels, health conditions and occupations.

## Conclusion

The current study offers psychometric evidence for the use of the PNSE-M to determine the levels of psychological needs satisfaction in exercise among Malay university students. All the items were kept with strong factor loadings. Based on Cronbach’s alpha, test re-test reliability, and discriminant validity, the translated PNSE-M lend itself to sufficient factor loadings, composite reliability, internal consistency. Furthermore, the scale demonstrated adequate measurement invariance (configural, weak, strong, and strict) as well as structural invariance (factor variance, covariance, and mean). These findings support the use of the PNSE-M to provide a reliable quantitative comparison between male and female.

## Supporting information

S1 Data (PDF)
