# Supplementary material for: Factorial validity and measurement invariance of the psychological need satisfaction in exercise scale across gender
Source: PLoS One. 2022 Jun 7;17(6):e0269155. doi: 10.1371/journal.pone.0269155 (PMC9173609; doi:10.1371/journal.pone.0269155)
Supplement: S1 Data — (PDF) [file pone.0269155.s001.pdf]

ID	Gender	Age	PNS1	PNS2	PNS3	PNS4	PNS5	PNS6	PNS7
1	1	20	3	3	3	3	2	3	3
2	2	20	6	6	3	6	4	4	4
3	2	20	2	4	4	3	6	4	4
4	2	20	6	6	3	4	5	4	4
5	1	21	4	3	4	6	4	4	4
6	2	22	2	4	4	5	6	4	5
7	2	20	6	4	3	4	4	4	6
8	2	20	3	4	4	3	4	4	4
9	2	21	6	6	4	4	6	3	6
10	1	21	3	4	4	4	6	6	3
11	2	23	3	4	4	5	6	4	4
12	2	19	6	6	4	4	6	4	4
13	2	19	3	3	3	4	3	4	3
14	2	19	6	6	4	5	4	5	5
15	1	19	3	4	5	3	4	4	4
16	1	26	4	5	5	5	5	5	5
17	2	19	6	6	6	6	6	6	6
18	2	22	3	6	5	4	6	4	4
19	2	21	3	4	4	3	3	4	4
20	2	21	5	6	6	6	6	6	6
21	2	21	3	3	4	3	4	3	4
22	2	20	4	4	5	5	6	5	5
23	2	21	4	4	4	5	5	5	5
24	1	21	4	3	4	4	4	4	3
25	2	21	4	4	4	4	4	4	4
26	2	21	1	6	3	2	3	3	1
27	2	21	6	6	6	6	4	6	5
28	2	19	5	5	6	4	5	6	5
29	2	21	5	4	4	4	4	5	4
30	2	21	4	6	5	4	5	6	5
31	2	20	5	5	5	3	4	4	4
32	2	23	4	3	3	4	3	4	4
33	2	20	4	4	3	3	4	4	4
34	2	20	4	4	4	4	5	4	4
35	2	20	4	4	4	4	4	4	4
36	2	19	4	6	5	6	4	6	4
37	1	18	2	2	2	3	2	2	2
38	1	19	5	5	6	4	5	4	5
39	2	20	5	5	5	5	5	5	5
40	1	23	4	4	4	4	4	4	4
41	2	20	4	4	4	4	4	4	4
42	2	20	2	2	2	2	2	2	2
43	2	24	4	4	4	4	4	4	4
44	2	20	6	6	6	6	6	6	6
45	2	20	1	4	4	2	4	1	4
46	1	20	5	5	5	4	5	5	6
47	2	20	3	3	3	3	3	3	3
48	1	21	4	4	4	4	4	5	4
49	2	20	4	5	6	6	6	4	4

50	2	20	4	4	5	5	3	2	2
51	1	21	4	4	3	4	4	5	3
52	2	21	4	4	5	4	3	5	4
53	1	22	3	3	3	3	4	4	3
54	1	20	3	4	5	6	5	4	4
55	2	21	4	3	4	4	5	3	1
56	2	21	5	5	4	5	3	5	4
57	2	19	4	4	5	3	5	4	6
58	2	20	4	3	4	5	4	3	4
59	2	22	4	3	3	3	3	3	3
60	1	21	5	5	6	4	5	5	5
61	2	22	6	6	5	6	5	6	6
62	2	22	6	5	6	6	6	5	5
63	2	20	3	4	5	5	4	3	3
64	2	19	3	4	5	5	6	4	4
65	2	19	4	4	5	4	6	6	3
66	2	21	4	5	3	2	1	3	4
67	2	20	4	3	3	4	4	5	4
68	2	22	4	3	3	4	3	4	3
69	2	21	5	5	5	5	5	5	5
70	2	21	4	4	5	4	5	5	4
71	2	20	4	5	4	5	4	4	4
72	2	20	5	5	5	4	5	5	5
73	2	20	5	5	4	3	4	3	5
74	2	22	5	4	3	5	2	4	4
75	2	21	5	5	5	5	5	5	5
76	2	20	4	4	4	4	4	4	4
77	2	20	4	3	4	6	3	4	5
78	1	20	6	6	3	3	6	4	4
79	1	20	5	5	5	5	4	4	4
80	2	20	5	5	5	4	5	6	6
81	1	20	5	5	5	4	6	5	5
82	2	20	5	1	4	4	4	6	4
83	1	19	4	4	3	4	4	4	4
84	1	20	4	4	4	5	3	4	5
85	1	20	2	1	4	1	1	1	1
86	1	20	4	4	4	4	4	4	3
87	1	20	6	6	6	5	5	5	5
88	2	20	6	6	6	6	6	6	6
89	2	20	6	6	6	5	6	6	6
90	2	20	4	6	5	5	5	6	5
91	2	19	4	4	4	4	4	3	4
92	1	20	4	4	5	4	3	3	4
93	2	18	6	6	6	6	6	6	6
94	1	22	5	5	4	4	5	5	4
95	2	20	4	4	3	3	3	3	4
96	2	19	2	3	4	4	4	5	4
97	2	19	4	4	4	3	4	4	4
98	1	21	4	4	3	2	4	5	3
99	2	19	4	4	4	5	5	5	5

100	2	20	3	4	4	3	5	4	3
101	2	21	4	4	5	6	4	3	4
102	2	21	5	5	4	4	5	4	5
103	2	20	5	5	4	6	4	4	3
104	2	20	5	6	4	5	4	5	3
105	2	21	2	5	5	1	5	3	3
106	1	24	3	5	5	3	5	4	4
107	2	20	2	4	4	1	5	4	4
108	2	24	2	3	4	3	4	4	4
109	2	22	3	5	5	2	5	3	5
110	1	21	2	4	4	2	4	3	3
111	1	21	3	5	5	3	5	4	4
112	2	21	1	2	3	2	3	4	3
113	1	23	2	3	4	2	4	4	4
114	1	21	1	4	4	1	4	3	3
115	2	20	2	5	5	2	5	3	5
116	2	22	4	4	4	4	4	4	3
117	2	19	3	4	3	3	4	3	2
118	2	19	4	3	3	3	4	5	2
119	1	21	3	4	3	3	3	3	3
120	2	21	5	5	5	4	5	5	5
121	2	22	5	5	5	5	5	5	5
122	2	19	4	4	3	4	4	3	4
123	2	19	4	6	5	3	5	4	5
124	2	19	6	4	5	6	3	6	5
125	2	21	6	5	5	5	5	5	4
126	2	18	4	4	6	5	4	4	6
127	2	18	3	4	5	3	4	4	5
128	2	19	5	5	5	5	5	5	5
129	1	19	2	2	3	3	4	3	4
130	2	22	2	2	2	3	3	3	2
131	1	19	2	3	3	3	3	3	3
132	1	19	4	4	4	3	4	4	4
133	2	20	4	4	4	4	3	4	4
134	2	19	4	4	4	4	4	4	4
135	2	20	4	4	3	3	3	4	4
136	2	24	4	4	4	4	3	4	4
137	1	22	4	4	5	4	5	4	4
138	2	21	5	6	6	4	6	4	6
139	1	18	3	1	1	2	1	3	1
140	2	22	4	3	4	4	4	4	4
141	2	22	3	4	4	5	5	4	4
142	2	20	6	6	6	4	6	5	6
143	2	20	5	5	4	5	6	5	4
144	2	21	3	4	3	4	3	3	3
145	2	20	3	3	3	4	3	2	3
146	1	20	3	4	3	2	3	4	5
147	2	20	4	3	3	2	3	4	4
148	1	21	3	3	3	4	3	2	4
149	1	21	3	3	2	2	2	2	2

150	1	19	3	3	3	3	2	3	4
151	2	20	3	5	4	4	3	4	4
152	2	20	4	3	4	2	2	4	3
153	1	19	6	5	6	5	5	6	6
154	2	20	4	3	4	4	5	4	4
155	2	23	3	3	2	2	2	1	2
156	2	20	4	4	5	5	5	5	4
157	2	18	5	5	5	5	6	6	6
158	1	23	6	4	3	5	5	2	2
159	2	21	5	5	4	5	4	5	5
160	2	21	2	5	3	2	4	5	5
161	2	19	1	3	3	2	4	2	3
162	2	20	5	4	6	5	4	5	4
163	2	23	4	6	5	5	5	4	4
164	2	20	6	1	5	6	5	6	6
165	2	21	5	5	5	5	5	5	4
166	2	23	4	4	4	3	3	3	3
167	2	20	4	4	3	5	4	5	3
168	2	24	4	3	3	3	4	3	3
169	2	22	1	1	1	1	1	1	1
170	2	22	4	4	4	4	4	3	4
171	2	23	5	5	5	4	5	5	3
172	2	22	6	5	5	5	5	5	5
173	2	22	4	6	6	5	4	4	4
174	2	21	4	3	5	4	3	5	6
175	2	18	3	4	4	3	4	3	4
176	2	21	3	4	4	4	3	4	4
177	2	23	4	4	5	5	4	4	4
178	1	20	4	5	6	5	5	4	5
179	2	19	6	4	5	6	4	6	4
180	2	20	3	4	5	3	5	3	3
181	2	23	4	4	3	5	5	2	2
182	2	20	4	4	3	5	5	2	2
183	1	19	4	4	4	3	4	4	4
184	2	19	4	4	4	4	4	4	4
185	2	19	4	4	4	4	4	3	4
186	2	19	3	4	2	3	4	3	4
187	2	20	3	4	5	5	4	4	4
188	2	19	4	3	4	5	4	4	5
189	2	20	3	3	3	4	3	3	2
190	1	19	6	6	4	6	5	4	5
191	1	19	4	4	4	5	4	5	6
192	2	18	6	5	4	4	4	3	4
193	1	19	5	6	6	6	5	5	5
194	2	18	5	5	5	5	5	4	4
195	1	19	5	5	5	6	6	6	6
196	2	19	6	6	5	5	5	6	6
197	2	19	5	5	5	4	4	3	3
198	2	19	5	6	6	6	5	5	5
199	2	19	3	3	3	2	3	4	4

200	2	19	5	4	4	4	5	4	3
201	2	19	3	4	3	4	4	4	4
202	2	19	2	3	2	3	4	4	3
203	2	19	3	4	3	4	5	4	4
204	1	19	4	5	4	3	3	4	5
205	2	19	3	2	4	4	4	3	4
206	1	20	4	3	5	3	5	4	2
207	1	20	2	4	3	2	3	2	3
208	2	19	3	3	2	4	3	3	4
209	1	19	4	4	4	5	4	3	4
210	1	21	4	3	4	4	3	4	4
211	2	21	3	3	3	2	2	2	3
212	2	21	5	4	5	5	4	3	4
213	2	22	3	4	4	3	4	4	4
214	2	22	5	5	6	6	6	5	6
215	2	22	3	3	4	3	3	3	4
216	2	22	4	4	4	3	4	3	4
217	2	22	4	5	4	4	4	4	5
218	2	19	3	6	6	1	6	6	6
219	2	19	5	6	6	6	5	6	5
220	2	21	6	6	5	5	5	6	5
221	2	19	3	3	3	3	2	3	2
222	2	21	6	6	6	6	6	6	6
223	2	21	3	3	4	4	2	3	4
224	2	20	3	4	4	3	4	3	4
225	2	19	4	4	4	4	4	4	4
226	2	23	5	5	5	5	4	5	5
227	2	24	5	5	4	5	4	5	4
228	2	19	4	4	4	4	4	4	4
229	2	19	6	6	6	6	6	6	6
230	2	20	3	4	5	4	3	3	4
231	2	20	2	4	4	2	3	2	4
232	2	20	3	3	3	3	3	3	3
233	2	21	1	1	1	2	1	2	2
234	2	20	4	4	4	4	5	4	4
235	2	20	3	3	3	3	3	3	3
236	1	21	4	4	4	6	4	4	5
237	2	20	4	4	4	4	4	4	3
238	2	20	4	4	4	4	4	4	4
239	2	21	5	5	4	4	5	5	5
240	2	21	3	3	3	3	3	3	3
241	2	21	5	5	5	5	5	5	5
242	1	21	4	4	4	4	4	4	4
243	2	19	3	3	3	3	3	3	3
244	2	22	3	3	3	3	3	3	3
245	2	24	4	4	4	3	3	3	3
246	2	20	3	4	4	4	3	4	4
247	2	20	3	4	3	4	3	3	3
248	1	24	5	6	5	6	5	5	4
249	2	23	3	4	5	4	4	5	3

250	2	23	3	4	5	2	5	2	5
251	2	24	2	4	4	1	4	3	4
252	2	18	4	4	4	3	4	4	5
253	1	23	4	4	4	5	4	5	4
254	2	19	2	6	3	2	6	2	4
255	2	19	5	4	4	5	4	6	4
256	2	18	3	5	6	3	4	4	6
257	1	19	6	6	6	6	6	6	6
258	2	20	4	4	3	4	6	5	5
259	2	19	3	4	4	4	4	4	3
260	2	24	4	4	4	4	3	3	3
261	2	19	4	5	4	3	4	5	4
262	2	19	4	4	4	4	4	4	4
263	2	19	3	3	3	3	3	3	3
264	2	22	3	6	6	4	6	5	5
265	2	20	3	6	6	1	6	3	6
266	2	23	4	4	4	4	4	4	4
267	2	22	3	4	3	4	4	4	4
268	2	21	4	3	4	3	3	4	4
269	2	20	5	4	5	5	5	5	4
270	2	20	5	6	6	6	6	5	5
271	2	19	4	5	5	4	5	4	5
272	2	21	3	3	4	3	4	3	3
273	2	20	3	4	3	4	3	3	3
274	2	18	3	3	4	3	4	3	4
275	2	19	4	4	5	3	5	4	5
276	2	20	4	5	5	3	4	3	4
277	2	20	4	4	4	4	4	4	4
278	2	22	6	6	6	5	6	6	6
279	2	21	4	3	5	3	4	5	6
280	2	21	4	3	5	3	4	5	5
281	1	21	3	6	3	1	3	4	3
282	1	24	3	2	4	2	3	4	2
283	2	21	5	5	5	5	5	5	5
284	1	20	4	4	3	2	4	4	4
285	1	20	4	5	5	5	4	5	4
286	1	21	4	4	5	5	4	5	4
287	1	21	4	4	4	3	3	3	3
288	2	19	2	3	3	3	3	3	3
289	2	19	5	5	5	5	5	5	5
290	2	22	3	3	3	3	4	3	4
291	2	19	4	4	4	4	4	3	4
292	2	21	4	4	3	3	4	3	4
293	2	22	3	4	4	3	5	4	4
294	1	20	3	3	3	3	3	3	3
295	2	19	6	6	6	5	6	5	5
296	1	21	5	5	4	3	4	5	4
297	1	21	4	4	5	5	5	5	5
298	1	19	4	4	5	5	4	5	5
299	2	22	4	6	6	4	5	5	6

300	1	21	4	4	5	4	5	5	5
301	1	21	3	3	4	4	4	5	4
302	1	21	3	4	4	4	4	5	4
303	2	21	4	4	4	4	4	4	4
304	2	21	1	6	4	5	4	4	4
305	2	20	5	4	6	4	5	4	5
306	2	21	6	5	5	4	4	6	4
307	2	21	5	5	5	5	5	5	5
308	2	21	5	3	5	5	5	4	5
309	2	19	3	2	3	2	3	2	3
310	1	19	4	4	3	4	4	5	4
311	2	19	4	4	5	4	6	4	5
312	2	21	3	2	3	2	4	2	3
313	2	22	4	4	5	4	4	4	4
314	2	21	5	5	5	5	5	5	5
315	2	21	4	4	4	3	4	3	4
316	2	20	3	3	3	3	3	3	3
317	2	20	3	6	6	5	4	5	5
318	2	19	3	6	6	5	6	6	6
319	2	19	3	4	5	6	4	4	4
320	2	19	3	3	4	4	3	4	5
321	2	20	4	5	6	5	6	4	6
322	2	20	4	4	4	4	4	4	4
323	2	20	4	5	4	4	4	5	5
324	2	18	3	3	4	3	3	4	3
325	2	20	4	4	3	3	3	3	4
326	2	19	5	5	5	5	6	5	4
327	2	18	2	3	3	3	3	3	3
328	2	22	3	6	4	3	5	5	4
329	2	20	4	4	5	5	6	3	4
330	2	20	4	4	4	2	3	2	2
331	2	20	3	3	3	3	3	4	4
332	2	19	5	5	6	4	5	5	6
333	2	19	3	3	1	1	3	3	1
334	2	19	3	4	3	2	3	2	3
335	2	19	4	4	4	6	6	5	5
336	2	19	5	4	4	4	4	4	4
337	2	19	4	4	4	4	4	4	4
338	2	19	4	4	4	6	4	5	5
339	2	20	4	3	3	4	3	4	3
340	2	21	4	3	4	3	4	4	3
341	2	20	3	4	4	2	4	4	4
342	1	19	4	4	4	3	4	4	4
343	1	23	4	4	3	4	4	3	4
344	2	20	5	5	5	5	5	6	5
345	2	20	4	4	4	4	4	4	4
346	2	21	4	4	4	4	4	4	4
347	2	20	1	4	4	2	4	3	4
348	2	21	6	6	6	3	4	4	2
349	2	20	4	4	4	5	4	5	5

350	2	20	6	6	6	6	6	6	6
351	2	21	2	3	2	2	3	2	2
352	2	20	4	5	5	4	6	5	4
353	2	20	3	4	5	4	5	4	4
354	2	23	3	4	4	3	5	3	5
355	2	21	4	3	3	4	3	3	3
356	2	21	4	5	5	4	5	4	5
357	2	19	3	3	3	3	3	4	2
358	1	19	3	5	6	4	5	4	3
359	1	22	5	5	5	5	4	5	5
360	1	21	6	5	5	6	4	6	5
361	1	23	2	4	5	2	1	2	4
362	2	19	3	3	4	4	5	3	4
363	1	20	6	6	6	6	5	6	5
364	2	19	4	4	4	4	4	4	3
365	1	19	6	6	6	6	6	6	6
366	1	20	6	6	6	6	6	6	4
367	1	24	5	5	4	5	4	5	4
368	2	24	4	4	4	4	3	4	4
369	1	19	3	5	5	2	6	6	5
370	1	19	4	3	4	4	3	4	3
371	2	22	4	3	4	4	4	4	4
372	1	19	1	6	6	3	6	6	6
373	2	19	4	4	5	5	5	5	5
374	1	20	6	4	3	4	4	3	3
375	2	19	3	6	6	4	6	5	5
376	2	22	5	5	5	5	5	5	5
377	2	24	4	4	5	4	5	4	4
378	1	21	4	4	4	4	4	4	4
379	1	21	2	2	3	3	4	3	4
380	2	21	3	4	4	4	4	4	4
381	2	21	6	3	3	5	4	5	3
382	2	21	4	6	5	4	5	4	3
383	2	21	4	4	4	4	4	4	4
384	1	21	5	6	6	5	6	5	5
385	1	19	5	5	5	5	5	5	5
386	2	19	2	5	5	5	5	4	5
387	2	19	2	3	4	3	4	4	3
388	2	21	3	2	4	2	3	4	2
389	2	23	6	6	6	6	6	6	1
390	2	19	5	5	5	5	5	5	5
391	1	19	4	4	6	5	4	4	4
392	1	21	3	4	5	3	5	3	4
393	2	20	5	5	5	5	5	5	5
394	2	19	5	6	6	6	6	6	6
395	2	19	6	3	5	4	3	5	3
396	2	19	4	4	4	4	2	5	3
397	2	18	4	4	3	5	3	5	3
398	2	22	4	4	4	4	2	5	3
399	2	19	5	5	5	4	5	3	5

400	1	23	3	4	4	2	4	3	4
401	2	19	3	6	6	1	6	1	4
402	2	20	4	4	4	4	4	4	4
403	2	24	6	3	3	6	4	5	3
404	2	19	4	6	6	3	6	4	6
405	1	24	3	4	4	4	3	4	4
406	2	23	6	6	6	6	5	6	6
407	1	22	5	6	6	6	5	5	5
408	1	21	5	5	5	5	5	5	5
409	1	19	4	5	5	5	5	4	4
410	2	19	5	2	2	2	2	5	2
411	1	21	4	4	4	4	6	6	6
412	1	20	6	6	6	4	5	4	4
413	2	20	3	4	4	3	4	3	4
414	1	20	6	5	4	5	5	4	4
415	1	20	3	3	6	5	5	4	5
416	2	20	3	3	2	3	4	4	3
417	2	22	4	4	4	4	4	4	4
418	2	20	4	5	4	4	4	5	5
419	2	20	3	4	5	2	4	2	4
420	2	20	5	5	6	6	5	6	5
421	1	21	3	2	3	4	5	5	4
422	2	20	6	5	6	6	6	6	6
423	1	20	2	2	2	2	2	2	2
424	2	20	5	2	2	5	2	5	2
425	2	19	6	5	5	5	6	6	5
426	1	19	4	4	4	4	4	4	4
427	2	19	5	4	4	4	4	4	4
428	2	20	4	4	4	4	3	4	4
429	2	19	3	3	3	3	3	3	3
430	1	20	4	4	4	3	4	4	4
431	1	20	3	1	1	2	1	3	1
432	1	21	4	4	5	4	5	4	4
433	2	24	4	4	4	4	3	4	4
434	2	20	5	5	5	4	4	5	5
435	2	20	4	3	3	3	4	5	5
436	2	21	4	4	4	4	5	4	5
437	2	19	3	4	5	2	2	3	2
438	2	19	4	6	3	3	5	5	4
439	2	19	1	1	3	1	1	3	1
440	2	18	6	6	6	6	6	6	6
441	2	19	4	5	4	4	5	5	4
442	2	19	5	1	1	5	1	5	3
443	1	22	4	5	6	4	5	5	4
444	2	19	2	5	5	2	5	2	5
445	2	25	4	5	4	4	3	6	4
446	2	20	4	3	3	4	4	4	4
447	2	19	3	4	5	4	4	4	5
448	2	19	5	5	5	5	5	5	5
449	2	19	4	4	4	4	4	4	4

450	2	19	6	6	6	6	6	6	6
451	2	19	5	5	5	5	5	5	4
452	1	19	6	6	6	6	6	6	6
453	1	25	5	6	5	6	5	5	5
454	2	20	5	6	6	6	6	6	6
455	2	20	6	5	6	5	5	5	6
456	1	20	6	5	4	3	4	4	4
457	2	25	6	6	6	6	6	6	6
458	2	19	4	3	6	5	5	5	4
459	2	23	5	4	4	5	6	6	6
460	2	19	5	6	6	5	6	6	6
461	1	20	1	2	3	4	2	3	4
462	2	19	4	2	6	5	4	3	2
463	1	19	6	4	3	2	5	6	4
464	1	20	4	2	4	2	6	4	2
465	2	20	4	3	2	5	2	5	3
466	2	20	5	3	2	6	2	5	1
467	2	20	6	3	5	1	4	2	5
468	2	19	3	5	2	5	3	5	4
469	2	20	5	3	2	1	1	6	3
470	2	20	2	6	1	3	2	4	1
471	2	22	3	5	4	2	3	1	3
472	1	20	2	6	4	3	2	1	4
473	2	20	1	1	2	3	1	2	2
474	2	20	4	4	4	4	4	4	4
475	2	20	4	3	3	4	4	3	4
476	2	23	6	3	3	5	3	3	3
477	2	19	4	4	4	4	4	4	4
478	2	19	3	4	5	4	5	4	5
479	1	19	3	3	4	3	3	3	3
480	1	19	5	5	5	5	5	5	5
481	1	19	4	4	4	5	5	4	6
482	2	19	5	4	6	5	5	5	5
483	2	19	4	4	4	4	4	4	4
484	2	19	2	3	4	3	4	2	4
485	1	19	4	5	4	4	4	4	4
486	2	19	5	6	6	6	5	5	6
487	2	21	3	3	2	2	4	2	2
488	1	19	6	6	5	5	5	5	6
489	1	19	6	6	6	5	6	5	5
490	2	24	4	3	5	4	5	4	4
491	2	19	5	6	5	6	5	6	5
492	1	21	5	5	4	5	4	5	5
493	2	21	5	5	5	5	6	5	5
494	2	19	6	6	6	5	5	5	5
495	2	21	4	5	5	4	4	4	4
496	2	21	5	4	5	5	4	5	5
497	2	19	4	4	4	3	4	3	4
498	2	21	4	5	4	4	4	5	5
499	2	20	2	3	3	2	4	5	4

500	2	20	2	4	4	2	3	3	3
501	2	20	3	3	1	1	3	3	1
502	2	20	5	5	6	4	5	5	6
503	2	20	4	6	6	3	5	5	6
504	2	20	3	4	4	4	5	4	3
505	2	20	6	6	6	6	6	6	6
506	2	20	3	3	3	3	3	3	3
507	2	20	4	4	4	4	4	4	4
508	2	20	6	6	6	6	6	6	6
509	2	20	3	6	6	3	6	2	4
510	2	20	2	5	5	1	4	2	2
511	2	20	4	4	4	3	4	3	4
512	2	20	4	4	4	3	4	3	4
513	2	24	4	4	4	4	4	4	4
514	2	20	4	4	3	4	5	4	4
515	2	19	5	5	5	5	5	5	5
516	2	20	4	4	5	4	5	4	5
517	2	20	4	5	5	5	5	4	4
518	2	22	3	4	4	2	4	3	3
519	2	21	5	6	6	5	6	5	6
520	2	19	4	5	5	3	5	3	6
521	2	19	5	6	5	4	5	5	5
522	2	22	3	3	3	2	2	2	3
523	2	19	4	2	4	4	4	4	2
524	2	19	4	4	4	4	5	4	5
525	2	19	4	5	4	4	5	4	5
526	2	20	3	3	4	1	3	1	1
527	2	20	3	1	1	2	2	3	2
528	1	19	6	6	6	6	6	6	6
529	1	19	3	1	1	3	2	3	1
530	1	20	5	5	5	6	5	5	5
531	1	20	5	6	6	6	3	6	1
532	2	20	5	5	5	6	5	6	5
533	2	20	4	4	5	5	5	5	4
534	2	20	4	4	3	3	3	3	3
535	2	20	5	5	5	5	5	5	5
536	2	20	3	3	1	1	3	3	1
537	2	20	5	5	6	4	5	5	6
538	2	22	3	3	3	3	3	3	4
539	2	21	4	3	4	4	3	3	3
540	2	20	4	4	4	4	5	4	4
541	2	20	4	4	6	6	3	5	5
542	2	21	6	6	6	6	6	6	6
543	1	23	6	6	6	6	5	6	5
544	2	19	4	5	4	4	4	5	5
545	2	23	6	6	6	6	6	6	6
546	2	19	4	4	4	4	5	5	5
547	1	19	6	5	5	6	5	6	5
548	2	19	6	5	5	6	5	5	6
549	2	19	5	5	4	5	5	4	5

550	2	19	6	5	6	5	4	5	6
551	2	19	6	5	5	5	6	5	4
552	2	19	6	6	6	6	6	6	6
553	2	18	5	5	5	5	4	5	5
554	1	19	5	4	4	5	4	5	4
555	2	21	6	5	6	5	5	6	5
556	2	18	5	6	6	5	6	5	6
557	2	22	6	6	6	6	6	6	6
558	1	19	4	5	5	4	6	4	4
559	1	19	3	4	4	2	4	3	4
560	1	19	4	4	3	4	4	4	3
561	1	18	4	3	3	3	4	3	4
562	1	25	5	3	4	6	4	5	4
563	2	20	6	5	5	6	6	6	6
564	2	20	4	4	5	5	6	6	6
565	2	20	4	2	4	4	3	4	3
566	2	20	4	4	4	4	4	4	4
567	2	18	3	4	5	4	5	4	5
568	2	19	3	3	4	3	4	3	3
569	1	19	3	3	3	2	2	3	4
570	2	19	3	3	4	4	4	4	4
571	2	20	4	6	6	5	6	5	6
572	2	20	3	4	3	3	3	4	4
573	2	19	6	6	5	5	5	6	5
574	2	19	3	6	6	1	6	6	6
575	2	19	6	6	5	5	5	6	5
576	2	19	4	5	4	4	4	4	4
577	2	19	5	4	5	5	4	3	4
578	2	19	4	4	4	3	4	3	4
579	1	21	6	6	6	6	6	6	6
580	2	19	3	3	3	3	2	3	2
581	2	20	5	5	5	5	5	5	4
582	2	19	4	3	3	3	3	4	4
583	2	19	4	4	3	4	3	4	3
584	2	19	5	6	6	6	6	6	6
585	2	19	4	4	4	4	4	4	4
586	2	19	5	5	5	5	5	5	5
587	2	19	3	4	5	5	6	5	5
588	2	19	3	1	4	3	3	3	3
589	2	19	2	1	1	2	1	1	1
590	2	19	5	5	5	5	5	5	5
591	2	19	5	5	5	4	5	4	5
592	2	21	4	5	5	4	5	4	5
593	2	18	4	4	4	3	4	4	4
594	1	19	2	2	3	3	4	3	4
595	2	19	4	4	4	3	4	4	4
596	2	21	5	6	6	5	6	5	6
597	2	23	4	4	4	4	4	4	4
598	2	22	5	6	6	6	5	5	4
599	2	20	5	6	5	6	5	5	4

600	2	20	3	3	3	3	3	3	3
601	2	20	4	3	4	4	3	4	4
602	1	24	3	4	4	3	5	3	4
603	1	19	3	3	3	3	2	3	2
604	1	24	5	6	5	5	6	5	5
605	1	24	4	4	4	5	5	3	5
606	1	20	4	4	5	6	6	4	5
607	1	20	6	6	6	6	6	6	3
608	1	24	6	1	6	6	6	6	1
609	1	20	4	4	4	4	4	4	4
610	1	23	6	5	6	6	6	6	5
611	1	22	5	5	5	5	4	5	5
612	1	23	4	4	4	4	4	4	4
613	1	23	4	4	3	4	3	3	4
614	1	23	6	6	6	5	5	5	5
615	1	19	5	6	6	6	5	5	5
616	1	20	3	3	4	2	4	3	3
617	1	19	6	6	6	6	6	6	6
618	1	19	3	4	4	3	4	4	4
619	1	21	6	4	3	5	4	5	5
620	1	20	6	5	5	5	5	5	4
621	1	22	3	4	3	4	3	4	5
622	1	19	2	4	6	1	6	5	1
623	1	22	5	4	5	5	4	4	5
624	1	21	4	4	4	3	4	4	4
625	1	19	4	4	4	4	5	5	5
626	1	19	5	4	4	4	4	5	5
627	1	19	4	4	4	5	5	4	5
628	1	19	4	3	5	4	5	4	5
629	1	22	2	2	1	2	3	3	2
630	1	20	6	5	4	5	5	6	5
631	1	24	5	4	4	4	6	5	5
632	1	21	3	3	3	4	4	3	4
633	1	23	5	4	6	5	6	4	5
634	1	20	4	4	4	4	5	5	5
635	1	19	4	4	4	5	5	4	5
636	1	20	5	4	5	4	5	4	5
637	1	19	5	4	4	5	6	5	4
638	1	24	2	2	3	3	3	3	3
639	1	20	4	5	3	5	2	4	5
640	1	20	6	4	5	5	4	5	6
641	1	20	3	3	4	4	3	5	3
642	1	19	3	4	4	5	5	4	4
643	1	18	6	5	5	3	4	5	5
644	1	19	4	5	4	5	4	6	4
645	1	20	4	4	4	5	4	5	4
646	1	24	4	5	5	3	5	5	5
647	1	19	5	4	4	5	4	4	5
648	1	19	4	4	4	3	4	3	4
649	1	19	5	5	4	5	5	5	4

650	1	19	3	3	4	5	5	4	3
651	1	19	4	4	4	5	4	4	4
652	1	19	6	4	5	6	6	6	4
653	1	19	3	4	3	3	4	3	4
654	1	20	3	3	4	3	4	3	4
655	1	19	4	3	4	4	3	3	3
656	1	19	4	4	4	4	4	4	4
657	1	20	4	5	5	5	5	4	4
658	1	20	4	4	4	4	5	4	4
659	1	21	4	3	3	3	4	4	4
660	1	21	4	4	4	4	4	5	5
661	1	23	4	4	4	5	5	5	4
662	1	20	4	4	5	3	5	4	5
663	1	20	4	5	5	3	5	3	4
664	1	22	4	4	4	4	5	5	5
665	1	23	4	6	4	5	4	6	4
666	1	21	4	4	4	4	4	4	4
667	1	21	5	5	5	5	5	4	4
668	1	21	4	4	4	4	4	4	4
669	1	23	6	6	6	6	6	6	4
670	1	20	3	5	5	2	6	6	5
671	1	20	6	6	6	6	6	6	6
672	1	20	4	4	4	4	4	4	4
673	1	20	6	6	6	6	6	6	4
674	1	23	3	5	5	2	6	6	5
675	1	21	5	5	5	4	5	5	5
676	1	18	4	4	5	5	3	3	4
677	1	18	4	6	4	4	5	4	4
678	1	20	5	4	4	4	4	4	4
679	1	18	1	2	3	2	1	1	1
680	1	22	4	4	4	4	2	4	2
681	1	19	3	4	4	3	3	3	4
682	1	19	4	4	4	4	5	5	5
683	1	19	6	6	5	5	5	5	5
684	1	19	4	5	4	5	4	4	4
685	1	19	4	4	4	5	4	5	4
686	1	20	4	3	4	4	3	4	3
687	1	20	3	3	3	3	4	3	3
688	1	19	4	4	3	3	3	3	6
689	1	21	6	6	6	6	6	6	6
690	1	20	4	3	5	5	5	4	5
691	1	21	4	2	4	3	4	5	3
692	1	21	4	5	4	4	4	5	4
693	1	24	4	4	4	4	4	4	4
694	1	21	4	5	4	5	6	4	5
695	1	22	3	4	4	2	3	2	4
696	1	23	2	4	5	2	3	2	3
697	1	20	3	4	4	2	5	2	4
698	1	19	2	3	3	2	4	2	3
699	1	19	2	4	3	2	3	2	3

700	1	22	3	2	4	3	3	4	2
701	1	22	3	3	3	4	3	3	2
702	1	24	3	3	4	3	4	3	3
703	1	19	4	3	4	3	4	4	4
704	1	20	5	5	5	5	5	5	4
705	1	20	5	4	5	5	5	5	5
706	1	20	5	5	5	5	5	6	6
707	1	23	4	4	4	4	3	3	4
708	1	19	5	4	4	4	4	4	5
709	1	20	2	2	3	3	3	2	4
710	1	20	4	4	4	4	4	5	2
711	1	20	2	1	2	3	2	2	3
712	1	24	4	4	4	4	5	5	6
713	1	20	4	3	4	5	4	4	5
714	1	22	4	5	4	3	4	5	4
715	1	25	3	4	4	4	3	4	4
716	1	19	5	4	4	3	4	3	4
717	1	25	4	4	3	4	5	5	5
718	1	22	6	6	5	6	5	6	5
719	1	24	4	4	4	3	4	4	4
720	1	23	5	5	5	4	5	4	5
721	1	20	5	5	4	4	4	4	5
722	1	20	3	3	3	4	4	3	4
723	1	20	3	4	4	3	3	3	4
724	1	24	3	2	3	3	2	2	2
725	1	21	3	3	2	2	2	2	2
726	1	19	3	3	2	2	2	2	2
727	1	19	4	4	4	3	4	4	3
728	1	20	5	4	5	4	4	5	4
729	1	19	4	4	5	4	3	3	4
730	1	20	5	6	6	5	4	5	5
731	1	20	4	3	4	3	3	4	3
732	1	21	5	4	5	4	4	4	3
733	1	26	3	3	3	2	4	3	3
734	1	20	5	5	4	4	4	3	4
735	1	19	5	4	4	4	5	5	6
736	1	20	5	5	4	4	5	6	6
737	1	21	2	4	3	5	4	6	4
738	1	20	4	4	4	4	4	4	4
739	1	20	4	4	4	4	3	4	4
740	1	20	4	4	4	4	4	4	4
741	1	21	4	4	4	4	4	4	5
742	1	22	4	4	4	4	4	4	4
743	1	20	4	4	4	4	4	4	4
744	1	20	5	4	5	6	5	4	5
745	1	20	4	4	4	4	4	4	4
746	1	21	5	5	5	6	5	5	5
747	1	23	6	6	6	6	6	6	6
748	1	22	4	6	6	5	6	5	5
749	1	23	5	5	6	5	5	6	6

750	1	21	5	6	4	4	5	4	5
751	1	23	4	3	5	3	4	3	5
752	1	23	6	5	5	6	5	5	5
753	1	24	3	3	4	2	2	3	4
754	1	25	4	4	5	5	5	4	4
755	1	24	4	4	5	4	3	4	4
756	1	24	5	5	4	4	4	4	5
757	1	20	6	3	4	5	4	4	4
758	1	21	3	1	1	3	1	3	1
759	1	23	4	4	4	4	3	3	3
760	1	20	4	5	5	4	4	4	5
761	1	23	6	5	5	6	5	5	6
762	1	20	5	4	5	5	5	5	5
763	1	20	3	5	6	3	5	4	4
764	1	23	4	4	4	4	2	4	3
765	1	21	6	2	2	6	2	6	2
766	1	20	3	2	3	4	4	3	4
767	1	21	3	3	4	4	4	4	4
768	1	23	4	6	6	6	5	5	5
769	1	23	5	4	4	4	5	6	6
770	1	21	6	6	6	6	6	6	6
771	1	23	6	6	6	6	6	6	6
772	1	21	6	6	6	6	6	6	6
773	1	22	6	6	6	6	6	6	6
774	1	23	4	4	4	4	4	4	4
775	1	20	5	6	6	6	6	6	6
776	1	20	3	3	4	4	3	4	4
777	1	20	5	5	5	5	5	5	5
778	1	19	4	4	5	3	5	3	5
779	1	19	5	5	5	5	5	5	5
780	1	19	5	5	5	5	5	5	5
781	1	20	4	5	5	4	5	4	5
782	1	19	2	3	4	4	5	3	4
783	1	24	4	4	4	5	4	5	5
784	1	19	2	3	4	4	5	3	4
785	1	20	5	5	5	5	5	5	5
786	1	21	5	6	5	4	5	5	5
787	1	19	5	6	5	4	5	5	5
788	1	24	2	2	3	3	4	3	4
789	1	24	5	4	4	5	4	4	5
790	1	20	5	4	4	4	3	4	5
791	1	19	4	4	3	4	3	4	4
792	1	19	6	6	5	6	5	6	5
793	1	19	4	5	5	5	5	5	4
794	1	19	5	4	5	5	5	4	4
795	1	19	4	3	3	4	4	4	3
796	1	22	5	5	5	4	5	5	5
797	1	19	5	5	4	4	5	5	5
798	1	22	5	5	5	5	5	5	4
799	1	19	5	5	4	5	4	5	5

800	1	24	5	5	5	5	4	4	5
801	1	19	2	2	3	3	4	3	4
802	1	19	5	4	4	4	4	4	4
803	1	19	5	5	6	5	5	5	5
804	1	19	4	4	2	3	1	4	2
805	1	19	2	4	3	2	2	2	4
806	1	23	4	2	5	6	4	3	5
807	1	21	4	5	4	4	4	5	5
808	1	21	4	5	5	5	5	5	5
809	1	24	3	6	6	3	6	3	6
810	1	21	1	1	1	1	3	2	1
811	1	21	4	1	1	5	1	4	1
812	1	21	4	4	3	4	3	4	3
813	1	19	3	2	3	2	3	1	2
814	1	20	5	4	4	5	4	5	6
815	1	21	5	5	4	5	5	4	4
816	1	19	4	4	5	4	4	5	3
817	1	23	3	5	5	2	6	6	5
818	1	19	5	6	4	6	4	6	4
819	1	19	3	4	4	3	3	3	3
820	1	19	5	4	5	4	5	4	4
821	1	19	4	1	4	3	4	3	4
822	1	19	4	3	3	2	3	2	4
823	1	23	4	4	3	4	4	3	5
824	1	20	2	3	2	3	3	3	1
825	1	22	5	4	4	4	3	4	3
826	1	23	4	5	5	4	5	3	4
827	1	21	4	6	6	4	6	4	6
828	1	23	4	5	5	4	5	4	5
829	1	22	3	4	4	3	4	3	4
830	1	20	4	5	5	4	5	4	5
831	1	22	3	5	5	1	6	3	6
832	1	20	3	5	5	3	5	3	5
833	1	22	4	5	5	4	5	4	5
834	1	23	4	5	4	3	4	3	4
835	1	20	3	4	3	2	3	2	4
836	1	21	4	1	2	3	2	3	3
837	1	21	4	3	3	4	3	3	3
838	1	20	3	3	4	4	4	4	4
839	1	20	4	6	6	4	6	6	5
840	1	19	4	5	6	4	6	6	5
841	1	20	5	5	5	5	5	5	5
842	1	20	4	5	4	4	4	4	3
843	1	21	6	4	4	6	4	6	5
844	1	20	5	4	5	5	5	5	5
845	1	21	5	5	5	5	5	5	5
846	1	21	4	5	5	4	5	4	4
847	1	20	4	4	4	5	5	5	5
848	1	22	5	5	5	5	5	4	5
849	1	20	4	4	5	5	5	5	5

850	1	20	5	5	6	6	5	6	5
851	1	20	4	4	5	5	5	5	5
852	1	20	4	5	4	3	5	4	5
853	1	20	3	4	3	4	4	5	3
854	1	21	6	5	5	6	5	6	5
855	1	20	3	4	3	4	5	5	6
856	1	24	5	5	5	5	5	5	5
857	1	20	4	5	5	6	5	5	5
858	1	19	4	3	4	3	2	3	2
859	1	19	6	4	5	6	4	6	4
860	1	19	4	4	5	5	5	5	5
861	1	19	4	4	4	5	4	4	4
862	1	19	6	5	5	5	5	5	5
863	1	19	4	4	4	3	4	4	4
864	1	19	4	4	4	4	4	4	4
865	1	19	5	4	6	5	6	5	4
866	1	19	4	4	5	5	4	3	4
867	1	19	6	5	5	5	6	5	5
868	1	19	3	4	4	5	4	5	3
869	1	21	4	2	4	4	2	4	2
870	1	21	4	4	4	4	4	5	4
871	1	21	4	4	4	5	5	4	5
872	1	21	3	4	4	4	4	3	4
873	1	21	4	4	4	4	4	4	4
874	1	21	4	4	4	3	4	4	4
875	1	21	4	4	4	4	5	5	4
876	1	23	1	2	3	3	4	5	4
877	1	20	4	4	4	4	4	4	4
878	1	22	5	4	4	3	3	5	3
879	1	19	5	6	5	6	5	4	5
880	1	19	3	3	3	3	3	3	3
881	1	20	3	3	3	3	3	3	3
882	1	19	5	4	5	5	4	5	4
883	1	19	3	5	6	4	6	4	4
884	1	22	3	4	4	3	4	3	4
885	1	24	6	5	4	5	5	4	5
886	1	20	5	5	5	5	5	5	5
887	1	24	5	5	5	4	5	4	4
888	1	22	5	5	5	5	5	5	5
889	1	21	4	4	4	3	3	3	3
890	1	19	5	4	5	4	4	6	4
891	1	19	5	6	6	4	6	5	5
892	1	19	5	5	5	5	5	5	5
893	1	23	5	6	6	6	5	5	5
894	1	21	3	6	6	4	6	4	4
895	1	19	4	5	5	5	4	5	4
896	1	23	3	4	4	2	3	2	3
897	1	24	4	5	5	3	5	4	5
898	1	19	6	5	5	4	6	4	5
899	1	20	5	5	4	4	4	4	4

900	1	22	4	4	5	4	4	3	4
901	1	21	2	4	5	3	4	3	2
902	1	19	5	3	5	4	4	4	4
903	1	19	3	3	3	4	3	4	3
904	1	23	4	3	3	3	3	4	3
905	1	19	5	4	4	6	5	4	5
906	1	19	3	4	3	4	3	3	3
907	1	20	5	4	4	5	4	5	4
908	1	19	1	5	3	1	4	6	1
909	1	20	1	1	1	1	1	1	1
910	1	19	5	5	5	5	4	4	4
911	1	20	6	6	6	6	6	6	6
912	1	25	5	5	5	4	4	4	5
913	1	23	6	6	6	6	6	6	6
914	1	19	3	4	4	2	4	4	4
915	1	20	6	5	6	6	6	5	5
916	1	20	4	5	6	5	5	4	5
917	1	20	4	6	5	4	6	5	2
918	1	20	6	6	6	6	6	6	6
919	1	22	5	6	6	5	6	5	6

PNS8	PNS9	PNS10	PNS11	PNS12	PNS13	PNS14	PNS15	PNS16	PNS17
3	6	3	3	6	6	3	4	6	5
4	4	4	4	4	4	6	4	6	4
4	4	6	6	4	4	3	4	3	4
4	6	6	4	4	4	4	3	4	4
3	4	2	3	4	6	6	6	4	6
6	6	3	4	4	4	6	6	4	2
4	3	6	4	3	4	4	3	4	3
4	4	4	4	6	4	2	4	3	4
2	6	6	4	4	6	4	4	6	6
4	4	4	5	4	3	4	4	3	6
3	6	4	3	4	6	4	4	4	6
4	3	2	6	4	6	4	6	4	6
4	5	3	5	3	6	2	3	4	2
3	4	6	4	3	5	4	5	5	4
5	4	4	4	4	5	4	5	4	4
4	4	4	4	4	4	5	5	5	5
6	5	6	6	6	6	6	6	6	6
6	4	4	3	3	6	5	5	5	6
4	3	4	4	3	3	3	4	3	3
6	6	6	6	6	6	6	5	5	5
4	4	3	4	3	4	3	4	4	4
5	5	5	5	5	5	5	5	5	6
4	5	4	4	4	5	5	4	5	5
4	4	4	4	4	4	4	4	4	4
4	4	5	4	4	4	4	5	5	5
6	2	2	2	3	6	6	3	4	6
6	6	6	6	6	6	6	6	6	6
5	5	5	5	5	6	5	5	5	6
6	5	5	4	4	4	3	5	4	5
5	4	4	4	4	5	5	6	5	5
5	5	5	5	4	5	4	6	5	5
4	3	4	5	5	4	4	3	4	5
5	5	3	5	3	6	4	6	4	6
4	4	4	4	2	4	4	4	4	4
4	4	4	4	4	4	4	4	4	4
6	6	6	6	6	6	6	6	5	6
2	3	2	2	2	2	2	2	2	2
3	3	3	3	4	6	5	5	5	6
5	5	5	5	5	5	5	5	5	5
4	4	4	4	4	4	4	4	4	4
4	4	4	4	4	4	4	4	4	4
2	2	2	2	2	2	2	2	2	2
4	4	4	5	5	5	5	5	5	5
6	6	6	6	6	6	6	6	6	6
3	1	1	4	2	4	3	3	3	6
5	5	6	6	4	6	5	6	5	6
3	3	3	3	3	3	3	3	3	3
5	4	3	3	4	3	3	3	3	3
5	5	3	3	4	3	4	5	3	4

4	5	3	4	5	4	6	4	5	4
4	6	4	5	6	5	3	4	5	6
6	6	4	3	3	6	4	6	5	6
4	3	2	3	5	4	4	3	4	4
4	5	5	5	4	6	3	1	1	4
2	4	3	3	4	2	3	4	3	3
6	6	2	4	3	6	4	5	4	5
6	4	2	1	3	4	4	3	5	6
3	3	3	5	4	6	2	2	1	3
3	4	4	3	3	3	3	3	3	3
4	5	4	5	6	6	5	6	4	5
5	6	5	6	5	5	5	5	5	5
5	5	5	6	6	6	5	6	6	5
3	3	4	5	6	4	3	2	2	1
5	6	1	2	3	4	4	5	5	3
4	2	3	4	3	5	5	6	3	1
5	4	3	6	4	3	2	3	4	5
4	5	6	4	4	5	5	4	5	3
5	6	5	5	5	5	2	6	2	6
5	5	4	4	5	5	5	4	5	5
5	5	4	5	4	5	6	6	6	5
4	4	4	5	4	5	4	5	5	5
5	4	4	4	4	5	5	5	5	4
4	4	5	4	4	3	4	3	4	3
4	4	4	5	5	3	4	4	5	6
5	5	5	5	5	5	5	5	5	5
4	4	4	4	4	4	4	4	4	4
6	3	3	4	6	6	3	3	3	3
3	3	4	4	3	4	5	4	5	5
5	4	3	3	1	4	4	4	3	4
5	4	5	5	5	6	5	4	5	6
4	4	5	5	6	5	3	4	5	6
6	6	6	6	4	6	4	6	4	6
4	4	5	5	5	5	5	4	5	5
4	4	3	5	4	5	4	3	4	5
1	1	1	1	1	1	1	1	1	1
4	3	4	4	4	4	4	4	4	4
6	6	4	3	3	3	3	2	2	2
5	6	5	6	5	6	3	6	5	6
4	4	4	4	3	3	4	5	5	3
5	5	6	5	5	5	5	5	5	4
4	4	3	4	3	4	4	4	3	5
5	4	4	5	4	4	4	5	4	4
6	6	6	6	5	6	6	6	6	6
4	5	4	5	5	4	4	5	5	4
5	4	4	3	3	4	4	3	4	4
4	4	3	4	4	5	5	4	4	3
4	4	4	4	4	4	4	4	4	4
2	5	3	5	3	3	1	3	3	4
5	5	5	4	4	5	6	5	5	5

5	4	4	3	3	4	4	3	4	4
4	5	3	2	2	4	1	3	2	3
4	4	4	5	4	4	4	5	3	4
4	5	5	3	2	4	4	5	5	4
4	2	2	2	1	3	5	5	4	5
5	5	3	5	1	5	5	5	5	5
6	6	4	5	2	6	5	5	5	6
4	5	3	5	1	6	6	6	6	6
4	4	3	4	2	5	5	5	5	5
5	5	4	4	2	6	5	6	5	5
5	5	4	4	2	5	5	5	5	5
5	5	4	5	3	5	5	6	5	6
3	2	2	3	3	4	4	3	4	3
5	5	3	3	1	5	5	5	5	5
5	5	4	5	1	5	4	5	5	6
5	5	4	4	1	6	5	5	5	6
4	5	5	4	3	4	4	4	4	4
3	3	2	4	3	5	5	5	5	4
4	4	3	1	4	3	3	2	5	4
4	5	3	5	2	6	4	4	4	4
6	6	4	5	4	6	5	4	5	6
4	5	5	5	5	4	5	5	5	4
4	3	4	4	3	5	4	5	5	4
5	5	4	5	4	6	4	4	5	6
4	5	4	6	6	6	3	6	5	5
5	5	5	5	5	6	5	5	5	5
3	4	3	3	1	5	3	4	6	5
6	5	3	3	2	4	4	4	4	4
5	5	5	4	5	5	5	6	4	5
3	4	4	4	3	4	2	1	3	1
2	2	4	2	3	4	3	4	1	3
3	3	3	3	3	3	3	2	4	3
4	5	4	5	3	6	6	6	6	6
3	4	4	3	3	3	3	3	3	4
4	4	4	4	4	4	4	6	5	5
5	6	6	6	5	6	5	5	4	5
4	4	4	4	4	4	2	4	2	5
5	4	5	4	5	5	4	5	4	5
4	3	3	3	3	3	3	5	5	3
6	6	4	3	3	6	1	6	2	2
4	4	4	4	4	4	4	4	4	4
3	3	4	4	4	5	5	5	4	4
6	6	5	5	5	6	6	6	6	6
5	5	5	6	4	5	6	4	5	6
4	4	4	3	3	3	3	3	3	3
3	3	2	2	2	3	3	4	4	4
4	4	4	3	4	5	4	3	4	4
3	2	3	4	4	3	2	3	2	3
3	4	5	4	4	4	5	4	4	3
2	2	3	3	4	4	3	4	4	3

3	4	4	4	3	2	2	2	3	4
5	5	3	3	3	5	5	4	5	5
4	2	4	3	3	2	4	3	4	3
6	6	6	5	6	6	5	6	6	5
3	3	4	4	4	4	4	4	4	4
4	5	3	4	4	4	4	5	3	5
6	6	3	4	5	6	5	5	5	6
5	4	4	4	6	5	6	6	6	6
4	3	3	4	5	6	6	4	2	3
5	4	3	4	4	5	4	5	4	6
5	4	3	4	2	4	3	3	5	5
5	6	1	3	2	6	4	3	4	5
6	5	5	4	4	6	5	6	5	6
4	4	4	4	4	5	5	4	5	5
6	6	6	6	5	6	4	4	3	6
3	3	3	4	3	4	3	4	5	4
3	3	3	4	4	3	3	4	4	4
4	4	4	3	3	5	4	4	4	4
3	2	2	2	2	2	2	2	2	2
1	1	1	1	1	1	1	1	1	1
4	4	3	4	3	4	4	4	3	5
5	5	4	4	4	5	5	4	4	4
5	5	5	5	5	5	5	5	5	5
5	5	4	5	5	5	4	5	6	4
6	5	5	5	5	6	3	5	5	6
4	4	4	4	3	4	4	4	4	4
3	3	3	4	4	5	5	6	5	5
5	4	4	3	4	5	5	4	5	4
4	4	4	4	3	4	5	5	6	4
6	6	6	4	6	6	4	3	4	6
5	3	3	6	3	6	4	6	4	6
4	4	6	3	1	4	2	5	3	6
4	4	6	3	1	4	2	5	3	6
3	4	4	3	3	3	4	3	3	3
4	4	4	4	6	6	4	6	4	6
4	3	3	3	3	4	3	3	4	3
5	5	5	5	4	4	5	4	4	4
5	5	4	4	5	5	5	4	3	2
6	4	4	5	4	4	5	5	4	4
3	3	3	3	2	2	2	3	3	3
4	6	5	6	4	5	5	6	4	5
5	4	5	5	6	5	4	6	5	5
3	3	3	2	2	3	4	4	3	3
6	6	6	6	6	5	5	5	5	6
4	5	4	5	6	6	6	5	4	4
5	5	4	4	5	6	6	5	5	5
6	6	6	5	5	4	4	5	5	4
3	4	4	4	3	3	3	3	3	3
5	6	6	5	5	5	4	4	5	5
3	3	4	3	3	3	3	4	3	4

3	4	5	4	3	4	4	5	4	4
3	4	3	4	5	4	4	4	4	4
2	4	3	2	5	4	2	3	2	5
4	4	3	4	4	3	4	5	4	3
2	2	3	4	3	2	3	4	3	2
3	4	4	3	3	2	3	4	3	3
5	4	4	6	4	4	4	3	3	5
4	3	3	4	3	4	4	3	2	3
3	4	3	3	3	4	3	4	3	2
5	4	4	4	4	4	3	5	4	5
4	3	4	3	4	3	4	3	4	5
3	3	3	3	3	2	2	2	2	2
4	3	3	5	2	5	3	4	4	3
4	4	4	4	3	4	4	4	4	4
6	6	5	5	5	6	5	5	6	4
5	5	3	3	2	6	5	6	4	6
4	3	3	4	4	3	4	3	3	4
5	5	4	4	5	4	5	4	5	4
6	6	6	6	4	6	6	6	6	6
5	6	6	6	5	6	5	5	6	5
5	6	5	5	6	6	5	5	4	5
4	4	3	4	3	4	3	4	3	4
6	6	6	6	6	6	6	6	6	6
5	4	6	5	4	5	4	6	4	5
5	5	3	4	3	5	4	5	4	5
4	4	4	4	4	4	4	4	4	6
6	6	5	5	4	6	4	5	4	5
4	4	5	5	4	4	4	5	4	5
4	4	4	4	4	4	4	4	5	5
6	6	6	6	6	6	6	6	6	6
2	2	4	5	4	3	3	3	5	4
4	4	1	3	1	3	3	5	3	3
3	3	3	3	3	3	3	3	3	3
2	2	1	3	2	2	2	1	2	1
4	5	4	4	4	6	5	4	5	5
3	3	3	3	3	3	3	3	3	3
4	4	5	5	4	5	4	5	4	5
4	4	4	4	4	4	4	4	4	4
4	4	4	4	4	4	4	4	4	4
4	4	5	5	5	5	5	5	5	5
3	3	3	3	3	3	3	3	3	3
5	4	4	5	5	4	4	4	4	5
4	4	4	4	4	4	4	4	4	4
3	3	3	3	3	3	3	3	3	3
3	3	3	3	3	3	3	3	3	3
3	3	3	3	3	3	3	3	3	4
4	4	3	3	4	4	4	4	4	4
4	4	4	3	3	3	3	4	4	4
5	6	5	5	4	5	4	4	5	5
6	3	4	5	4	4	4	4	5	5

5	5	2	5	2	5	4	5	5	5
4	4	3	4	4	3	4	3	4	3
4	3	4	4	3	3	4	5	4	6
3	4	5	4	4	3	4	5	4	4
6	6	3	2	2	6	6	6	4	6
6	3	6	5	5	5	3	6	2	2
6	6	4	6	4	6	4	4	6	6
6	6	6	6	6	6	6	6	6	6
4	4	4	3	3	3	4	5	6	5
3	3	2	3	4	3	4	3	4	5
4	4	4	4	3	4	4	3	4	4
4	5	4	4	4	3	5	4	5	6
4	4	4	4	4	4	4	4	4	4
3	3	3	3	3	3	3	3	3	3
4	3	4	4	4	3	5	4	6	4
6	6	3	5	1	6	6	6	6	6
4	4	4	4	4	4	4	4	4	4
4	4	4	4	4	4	5	5	5	5
3	4	3	3	4	3	3	4	4	3
6	6	5	5	5	5	5	5	5	5
5	6	5	6	5	6	6	6	6	6
4	5	4	4	4	5	5	5	5	5
3	3	2	3	3	3	3	3	3	3
3	2	3	3	3	3	3	3	3	4
3	3	3	4	3	1	3	5	4	4
4	4	4	4	4	4	4	4	5	4
5	5	3	5	3	6	6	6	6	6
5	5	4	5	4	5	5	5	5	5
6	6	6	6	6	6	6	6	6	6
5	4	3	4	4	5	5	4	4	6
5	4	4	5	4	5	4	4	6	4
2	3	4	5	3	5	3	4	5	6
3	4	3	4	3	3	2	3	4	3
6	6	6	6	5	5	5	5	5	5
4	4	3	3	4	5	3	4	4	4
4	4	4	4	4	4	4	4	4	4
4	4	4	4	4	4	4	4	4	4
5	4	4	2	3	3	3	4	3	3
3	3	2	3	3	3	3	3	3	3
6	6	6	6	5	5	5	5	5	5
3	4	3	4	3	4	3	3	3	3
3	3	4	3	4	4	4	4	4	4
3	3	4	4	4	4	3	3	3	3
4	4	3	5	4	4	5	4	5	4
3	3	3	3	3	3	3	3	3	3
5	5	6	6	5	6	6	6	6	6
5	5	5	3	4	3	6	5	5	6
5	4	5	5	5	5	5	5	5	5
5	5	4	4	4	6	4	5	5	4
6	3	4	5	4	5	5	6	6	5

5	4	4	5	4	6	5	5	5	5
4	4	4	4	4	4	5	4	5	5
5	4	4	5	4	5	4	4	5	5
4	4	4	4	4	4	4	4	4	4
5	4	4	4	4	4	4	5	5	5
5	5	4	4	3	5	6	6	6	5
3	3	5	4	4	3	5	5	6	6
5	5	5	5	5	5	5	5	5	5
5	6	5	5	5	5	3	5	5	5
2	2	2	2	3	2	4	2	3	3
5	5	5	5	5	6	3	5	4	6
4	4	3	4	3	4	4	5	5	5
4	5	4	3	3	5	5	6	6	6
4	6	4	6	4	6	5	6	5	6
4	5	5	5	5	5	5	5	5	5
4	4	4	4	4	4	4	4	4	4
5	4	3	3	3	5	5	5	5	5
5	5	5	5	5	5	5	5	5	5
6	6	5	6	5	6	6	6	6	6
6	6	3	6	6	6	6	6	6	5
4	3	4	5	4	4	3	4	4	5
6	6	5	6	5	6	6	6	6	6
5	5	4	5	4	4	4	5	4	4
5	4	5	4	5	5	5	4	5	5
4	4	3	3	4	3	4	3	4	4
4	4	2	3	3	4	4	3	4	3
4	4	5	5	6	5	5	5	5	6
3	3	2	3	3	3	3	3	3	3
5	5	5	3	2	6	6	5	4	5
4	4	5	5	5	6	6	5	6	6
4	4	2	3	2	4	4	2	5	5
3	3	3	4	3	4	4	4	4	4
4	4	4	6	4	4	5	4	4	4
4	4	3	4	2	6	1	6	4	6
3	3	2	3	2	3	4	3	3	3
6	6	6	6	5	6	6	6	5	6
4	4	4	4	4	4	4	4	4	4
4	4	4	4	4	4	4	4	4	4
5	4	4	4	4	4	4	4	4	4
4	4	4	3	3	3	3	3	3	3
4	5	4	4	3	4	4	4	4	4
3	3	3	4	2	3	4	3	4	4
4	4	4	4	4	4	4	4	4	4
3	3	4	3	4	4	4	3	4	3
5	4	5	4	5	5	4	4	5	6
4	4	4	4	4	4	4	4	4	4
4	4	4	4	4	4	4	4	4	4
5	5	3	5	5	6	4	2	5	6
6	6	4	5	4	5	6	6	6	6
5	5	5	5	5	5	4	5	5	6

6	6	6	6	6	6	6	4	6	6
3	3	2	3	2	2	2	3	3	2
2	2	4	4	4	5	6	6	5	6
4	4	4	4	3	5	5	4	5	4
5	4	3	3	3	5	5	3	5	5
4	3	3	2	3	5	4	3	3	4
4	4	3	5	4	5	4	5	4	4
3	4	2	2	3	4	3	2	3	4
4	4	4	5	4	4	5	4	6	4
6	5	6	6	5	6	5	5	5	6
6	6	6	6	6	5	5	6	5	6
5	5	2	4	2	5	4	2	4	5
3	3	3	4	3	5	3	4	3	4
6	6	6	6	6	5	5	6	5	6
5	5	4	4	4	5	4	4	5	5
6	6	6	6	6	6	6	6	6	6
6	6	6	6	5	6	6	6	5	6
5	5	5	5	5	5	4	5	4	5
5	5	4	5	4	4	4	5	4	5
5	4	5	4	5	6	1	6	5	4
4	4	4	5	4	5	3	4	5	4
5	6	5	5	4	4	5	5	5	5
1	1	1	1	1	4	6	6	6	6
5	5	5	5	4	5	5	5	5	5
5	2	4	5	3	3	5	5	6	3
4	3	4	4	4	3	5	4	6	4
5	5	5	5	5	5	5	5	5	5
5	5	4	4	3	5	4	4	5	5
4	4	4	4	4	4	4	4	4	4
3	4	4	4	3	2	2	1	3	1
4	4	3	4	3	4	4	4	4	4
4	5	3	4	4	4	3	5	5	4
5	5	4	5	5	5	4	4	4	5
4	6	4	6	4	6	4	6	4	6
5	5	4	6	5	5	5	6	5	5
6	6	6	6	5	5	5	6	6	6
5	6	6	5	5	5	5	5	5	5
4	4	3	4	4	3	5	5	5	5
4	3	5	3	4	2	4	3	5	2
6	6	6	6	6	6	6	6	6	6
4	4	4	4	3	4	4	4	4	5
3	6	4	5	4	6	4	6	6	6
4	4	4	4	3	4	5	4	5	5
5	5	5	6	6	6	6	5	5	5
6	6	6	6	6	6	6	6	6	6
4	5	3	6	4	6	3	6	4	6
4	3	4	4	3	6	3	6	6	6
5	5	5	5	5	5	3	5	4	5
4	3	4	4	3	6	4	6	6	6
5	5	5	5	5	5	5	4	5	5

4	4	3	4	3	5	4	5	4	5
3	1	1	6	1	6	6	1	6	6
4	4	4	6	4	6	5	6	6	6
5	5	5	5	3	5	3	5	3	5
6	6	3	6	3	6	6	4	6	5
5	5	4	3	4	5	4	4	3	3
6	5	6	6	6	6	5	6	6	5
5	5	6	5	5	4	4	5	4	4
5	5	6	5	5	5	6	6	6	5
4	4	5	5	5	4	4	5	5	4
5	5	5	5	5	5	2	5	5	5
6	6	4	6	6	5	6	5	6	5
4	6	5	6	4	6	6	6	6	5
4	3	3	4	3	4	4	4	5	5
4	5	4	5	4	5	4	6	4	5
6	3	3	5	2	5	6	5	5	3
3	3	4	3	3	3	3	3	3	4
4	4	4	3	3	4	4	3	3	4
4	4	5	3	3	4	4	3	2	3
4	4	3	4	2	5	5	2	5	5
5	5	5	6	5	5	6	5	5	5
4	4	4	4	3	4	5	5	6	5
5	5	5	6	6	6	6	6	6	6
2	2	2	2	2	2	2	2	2	2
5	5	5	5	5	5	2	5	2	5
5	5	6	6	6	6	5	5	5	5
4	3	4	4	4	4	4	4	5	5
5	5	4	4	4	5	4	5	4	5
3	4	4	3	3	3	3	3	3	4
3	3	3	3	3	3	3	3	3	3
4	5	4	5	3	6	6	6	6	6
6	6	4	3	3	6	1	6	2	5
5	4	5	4	5	5	4	5	4	5
4	4	4	4	4	2	4	2	5	5
4	3	3	4	3	3	4	5	5	5
4	4	5	5	5	6	6	6	5	5
4	4	5	4	4	5	4	5	5	4
5	4	2	3	1	5	4	3	3	4
6	6	6	6	3	6	3	4	4	6
3	3	3	3	1	6	1	6	1	6
6	6	6	6	6	6	6	6	6	6
4	4	4	4	3	4	4	4	4	4
6	6	4	4	6	6	3	3	1	6
4	4	4	5	4	5	6	6	5	6
5	5	3	4	2	5	5	3	5	5
5	4	5	5	4	5	4	5	3	2
4	4	4	3	5	5	4	4	4	5
5	4	4	4	4	5	4	5	5	5
5	5	5	5	5	5	5	5	5	5
4	4	4	4	4	4	4	4	4	4

6	6	6	6	6	6	6	6	6	6
5	5	5	5	4	5	5	4	5	5
6	6	6	6	6	6	6	6	6	6
6	5	6	5	6	4	5	4	5	4
6	5	5	5	6	5	6	5	5	6
5	5	5	6	6	5	4	5	6	5
5	6	6	5	6	5	4	4	5	6
6	6	6	6	6	6	6	6	6	6
6	6	4	3	4	5	5	6	6	6
5	6	5	6	4	6	4	5	4	5
5	6	6	6	5	5	6	6	6	6
5	5	3	5	6	3	5	3	2	6
6	5	3	2	1	6	2	5	2	5
2	5	3	5	3	5	2	5	2	1
4	6	4	2	1	6	3	5	3	5
2	2	5	5	4	5	2	5	2	5
3	6	1	5	2	6	4	1	3	1
3	1	4	2	5	3	1	4	2	6
6	3	6	4	2	1	4	6	4	6
4	2	1	5	4	3	2	5	4	3
4	6	4	2	4	6	4	2	3	6
3	4	6	3	6	3	6	1	6	4
2	5	3	2	1	6	4	3	6	2
2	2	1	3	2	2	2	1	2	1
4	4	4	4	4	4	4	4	4	6
4	4	4	4	5	5	4	5	4	3
5	5	5	5	5	5	3	6	3	5
4	4	4	4	4	4	4	4	4	4
5	5	4	5	4	5	5	5	5	5
4	4	3	3	3	4	4	4	4	4
5	4	4	5	4	5	5	5	5	5
6	4	4	5	3	4	4	4	5	4
6	6	5	5	5	6	5	6	5	6
4	4	4	3	3	4	4	4	4	4
5	4	2	2	2	4	4	4	4	5
4	4	5	4	5	4	4	4	4	4
5	5	6	5	6	6	6	5	6	6
3	3	3	3	3	3	3	3	3	3
5	5	5	5	5	6	5	5	6	5
5	6	5	5	6	5	5	5	5	6
5	5	4	4	5	4	4	5	4	6
4	5	4	5	5	6	5	4	6	5
4	5	4	4	4	5	5	5	5	4
6	5	5	5	5	5	6	6	6	6
5	6	5	5	5	5	5	6	6	5
4	4	4	5	5	5	5	5	6	6
4	4	4	5	4	5	4	5	4	4
4	4	4	3	4	4	3	3	3	4
5	4	5	4	5	5	5	4	5	5
4	3	3	4	4	3	4	4	3	3

3	3	4	3	2	3	4	4	4	4
4	4	3	4	2	6	1	6	4	6
4	4	4	6	4	4	5	4	4	4
6	6	3	5	3	6	6	6	6	6
4	4	4	4	5	5	6	4	5	4
6	6	6	6	6	6	6	6	6	6
2	2	2	2	2	4	4	3	3	3
4	3	3	3	4	3	4	4	3	3
6	6	6	5	5	6	6	6	6	6
6	4	4	4	4	4	5	5	5	5
4	2	2	2	2	3	4	2	4	4
4	4	4	4	4	4	4	6	4	6
4	4	4	4	4	4	4	6	6	6
4	4	4	4	4	4	4	4	4	4
4	5	6	5	4	4	4	5	6	4
6	5	5	5	4	6	5	6	6	6
5	5	5	5	4	5	5	5	5	5
4	4	5	5	3	4	3	4	4	5
5	5	3	3	1	3	4	5	5	5
6	6	5	5	5	6	6	6	6	6
6	6	4	3	6	5	6	6	6	6
5	5	4	4	3	5	5	5	4	5
4	4	2	4	2	3	3	3	3	3
4	4	4	4	4	2	4	4	4	3
5	5	4	4	4	6	5	4	5	5
5	5	4	4	3	4	5	5	5	5
6	4	4	3	2	6	4	6	6	6
3	3	3	3	2	3	2	3	2	3
6	6	6	6	6	6	5	6	6	6
3	3	3	3	3	3	1	3	1	3
5	5	5	5	5	6	5	6	6	5
6	6	5	5	5	5	6	6	4	3
6	6	6	6	5	6	5	6	6	6
5	5	5	6	6	6	4	4	5	4
3	3	3	3	4	4	5	5	5	4
5	6	6	5	6	5	5	6	5	6
4	4	3	4	2	6	1	6	4	6
4	4	4	6	4	4	6	4	4	4
4	3	3	3	4	3	4	4	3	3
4	4	4	4	4	4	4	3	4	4
5	5	4	4	4	5	5	5	4	5
6	6	6	3	5	3	6	6	6	6
6	6	6	6	6	6	6	6	6	6
5	5	5	6	6	5	5	6	6	6
5	4	5	4	5	5	5	4	5	5
6	6	6	5	5	6	6	6	6	6
5	5	5	6	6	6	6	6	6	6
6	6	5	5	6	4	5	6	4	5
5	5	4	5	5	4	5	6	5	6
4	5	5	5	4	5	5	5	4	5

5	4	5	4	5	5	6	5	5	4
5	5	5	5	4	5	5	4	5	6
6	6	6	6	6	6	6	6	6	6
4	5	4	4	4	5	5	5	5	4
6	6	6	6	6	6	6	6	6	6
4	4	5	5	6	5	4	5	6	5
6	6	5	6	5	6	6	6	6	6
6	6	6	6	6	6	6	6	6	6
6	6	4	6	4	6	4	6	6	6
5	5	2	3	3	4	4	6	5	5
4	4	4	4	4	4	4	4	3	3
3	3	4	3	4	3	4	3	4	3
6	5	5	6	5	6	5	6	5	6
6	6	5	5	5	5	6	6	6	6
5	4	4	6	6	6	4	6	4	6
3	4	4	4	4	4	4	4	4	4
4	4	4	4	4	4	4	4	4	4
5	5	4	5	4	5	5	5	5	5
4	4	3	3	3	3	3	3	3	3
5	4	3	4	3	5	3	2	3	3
3	3	3	2	3	3	3	3	3	3
6	6	5	6	5	6	6	6	6	6
3	3	4	3	3	4	4	3	4	4
5	6	5	5	6	6	5	5	4	5
6	6	6	6	6	6	4	6	6	6
5	6	5	5	6	6	5	5	4	5
5	5	5	4	4	5	4	5	4	4
4	3	3	5	2	4	3	4	4	3
3	3	4	4	4	3	3	4	4	3
6	6	6	6	6	6	6	6	6	6
4	4	3	4	3	4	3	4	3	4
5	5	4	5	5	4	5	4	4	5
4	4	4	3	3	4	2	5	4	4
3	3	3	3	3	3	3	4	3	4
6	5	5	4	4	5	5	5	6	6
4	4	4	4	4	4	4	4	4	4
5	5	5	5	5	5	5	5	5	5
6	6	4	5	2	4	5	5	4	5
3	3	3	4	2	3	3	4	4	4
1	1	1	1	2	1	2	1	1	1
5	5	5	5	5	5	5	5	5	5
5	4	5	4	5	4	5	4	5	5
4	4	4	4	4	4	4	4	4	4
5	5	4	4	4	4	4	4	4	4
3	4	4	4	3	2	2	1	3	1
3	4	4	4	3	4	4	3	4	3
6	6	5	5	6	6	6	6	6	6
4	4	4	4	4	4	4	4	4	4
3	4	3	4	4	4	3	3	3	4
4	3	3	4	4	3	3	3	3	4

3	3	3	3	3	3	3	3	3	3
4	4	4	4	4	4	4	3	3	3
6	6	3	4	3	6	5	4	5	6
3	2	3	4	3	2	3	4	3	2
4	5	6	5	5	4	5	4	4	5
4	4	5	4	4	3	4	5	4	5
5	4	5	4	4	6	4	4	4	6
6	6	6	4	6	6	3	6	3	6
6	6	6	6	6	6	1	6	6	6
4	4	4	4	3	4	4	4	4	4
4	4	5	6	5	5	6	6	5	5
6	6	5	6	4	6	5	5	4	6
4	3	4	4	4	4	4	4	4	4
3	4	4	4	3	3	3	4	3	4
5	5	5	5	4	5	5	5	5	5
5	5	5	5	5	6	6	6	6	5
3	3	3	3	2	3	3	2	3	2
6	5	5	4	4	6	6	6	6	6
4	4	4	4	4	4	4	4	3	4
6	5	5	5	5	6	5	4	4	6
5	6	6	5	5	5	5	6	5	6
3	4	3	4	4	3	4	4	5	3
6	6	6	5	4	4	4	5	5	5
6	6	5	6	6	6	5	4	4	5
4	4	3	4	4	3	3	4	3	4
5	6	5	5	5	6	5	5	5	4
3	3	4	4	3	5	5	5	4	4
4	4	4	5	5	5	5	5	5	5
5	4	3	4	3	5	5	4	4	5
4	3	3	3	2	5	2	4	4	5
4	5	6	5	4	5	6	5	4	5
4	4	5	6	6	5	4	5	6	4
4	4	3	4	4	3	3	3	4	4
5	6	4	5	4	6	5	5	6	5
5	5	4	4	5	5	5	4	4	5
5	4	4	4	5	4	5	5	5	4
3	5	5	4	4	3	3	6	2	4
3	4	4	5	6	5	4	3	2	3
3	3	3	3	3	2	2	2	2	2
4	6	5	6	5	6	4	6	4	5
5	5	5	4	4	5	5	5	4	5
5	4	6	5	5	6	6	4	5	3
4	3	3	3	3	4	4	4	5	4
4	6	5	4	1	6	4	3	4	5
5	4	6	4	5	4	4	5	4	5
6	6	4	4	4	6	4	4	5	6
6	6	6	5	4	4	4	5	5	4
4	5	4	1	1	1	4	4	4	4
4	3	4	4	4	4	3	4	4	4
5	5	4	5	5	5	5	5	5	4

3	4	3	4	5	3	4	4	3	4
5	5	5	4	5	4	4	4	5	5
6	6	6	6	5	6	4	6	4	4
3	3	4	4	3	3	4	4	3	4
3	4	4	4	3	3	4	4	3	4
4	4	3	4	3	4	3	4	3	4
4	5	5	5	4	5	5	5	4	5
4	4	4	4	5	4	5	5	5	5
4	4	5	4	5	5	5	5	5	4
4	4	4	4	4	4	4	5	4	5
5	5	5	3	3	3	3	3	3	5
5	5	5	3	3	3	3	3	4	5
4	3	3	4	3	3	5	4	5	4
4	4	4	3	3	4	4	4	5	4
5	5	5	4	3	3	3	3	3	5
6	6	6	6	4	6	6	6	6	6
4	4	4	4	4	4	4	4	4	4
4	5	4	5	4	5	5	5	5	4
4	4	4	4	4	4	4	4	4	4
6	5	5	5	5	6	5	5	5	6
5	4	3	4	5	6	6	5	4	4
6	6	6	6	6	6	6	6	6	6
4	4	4	4	4	4	4	4	4	4
6	5	5	5	5	6	5	5	5	6
5	4	3	4	5	6	6	5	4	4
5	5	4	5	4	5	4	5	5	5
4	5	4	3	3	4	5	5	4	4
5	6	5	5	5	4	4	5	3	4
4	4	4	4	4	4	4	4	4	4
1	6	1	6	1	6	1	1	3	6
4	4	4	4	4	4	4	4	4	5
4	4	3	3	3	4	4	4	3	4
5	5	5	5	5	5	5	5	5	5
5	5	6	5	5	5	5	5	5	5
4	4	4	4	5	4	4	5	4	4
4	4	4	4	5	5	5	5	4	4
4	3	4	4	4	4	3	2	4	2
2	3	3	3	3	2	4	3	3	3
5	5	4	4	4	4	4	4	4	4
6	5	6	6	5	6	6	6	6	6
5	5	3	6	5	5	4	5	6	4
4	4	3	5	4	3	4	4	3	5
5	5	4	5	4	6	4	4	4	6
4	4	3	4	4	4	4	3	3	4
4	5	6	4	5	5	4	5	5	4
5	5	2	4	2	5	4	3	4	5
4	4	2	5	2	5	5	4	4	5
5	4	2	3	2	4	5	2	4	3
4	4	2	3	2	3	4	3	4	3
4	4	2	3	2	2	3	2	4	3

6	6	3	4	4	3	4	4	4	6
3	3	2	3	3	2	2	3	3	2
2	3	2	2	2	3	4	3	3	4
3	4	3	3	3	4	3	4	4	3
4	5	5	5	5	5	5	5	5	5
5	5	5	5	5	4	5	5	4	5
6	6	6	6	6	5	5	6	6	6
4	4	3	4	4	4	4	3	4	4
4	4	5	5	4	4	5	5	5	4
4	4	3	2	3	4	3	4	3	2
6	6	4	6	3	6	4	5	4	6
3	3	4	4	4	3	2	2	2	3
5	6	5	4	5	6	5	6	5	4
3	4	5	5	4	4	3	4	5	3
4	3	4	5	4	5	4	3	4	5
4	3	3	3	3	3	3	3	4	4
3	4	4	3	4	3	3	4	4	3
4	4	3	4	3	5	3	4	5	4
5	5	6	6	6	5	5	6	5	5
6	5	4	4	5	5	3	3	4	4
4	5	4	5	4	5	5	5	5	5
4	4	4	4	3	3	4	5	4	5
4	4	3	4	4	4	3	4	3	3
3	4	4	3	3	4	4	3	4	3
3	3	3	3	2	2	2	2	2	3
2	2	2	2	2	2	2	2	2	2
3	2	2	2	2	2	2	2	2	2
3	3	4	4	4	3	3	4	3	3
4	4	5	5	6	6	5	6	5	5
4	4	5	4	3	3	4	4	5	5
6	6	4	4	5	5	5	6	6	4
5	5	4	4	3	4	4	4	5	5
4	4	5	4	5	4	4	4	5	4
2	2	3	4	3	2	3	3	3	2
3	5	5	5	4	4	4	4	5	5
5	5	4	4	5	6	4	5	5	4
5	4	5	4	3	4	5	3	4	6
5	3	3	5	2	3	4	4	5	4
4	4	4	4	4	4	4	4	4	4
4	4	4	4	4	4	4	4	4	4
4	4	4	4	4	6	5	5	5	6
6	6	4	4	5	6	4	4	4	6
4	4	4	4	4	4	4	4	4	4
4	4	4	4	4	4	4	4	4	4
6	5	5	5	5	4	4	4	4	4
5	4	3	4	5	5	5	5	5	5
5	5	5	5	5	5	5	5	5	5
6	6	6	6	6	6	6	6	6	6
6	6	6	6	5	5	6	6	5	6
5	5	5	5	5	5	5	5	5	6

4	4	5	4	4	5	4	4	4	4
4	3	5	3	5	4	4	3	5	4
6	6	5	6	5	6	5	6	5	6
4	3	3	4	3	4	4	3	2	3
5	5	5	5	4	6	4	4	5	6
4	5	4	5	5	4	5	6	6	5
4	5	4	6	5	4	5	5	4	3
6	6	6	6	6	4	6	4	5	4
3	3	3	6	3	6	1	3	3	3
3	4	4	3	3	4	3	4	4	4
5	4	5	4	4	5	5	4	5	5
4	5	6	6	5	4	4	5	5	5
5	6	6	6	5	5	5	5	4	4
5	5	4	4	3	6	6	6	5	6
4	5	4	4	3	4	3	5	5	5
5	5	5	5	4	5	2	4	2	4
3	4	5	5	4	4	4	4	4	4
4	4	4	4	5	5	5	4	4	4
5	4	4	4	4	5	5	4	4	5
6	6	5	5	5	5	6	6	5	4
6	6	6	6	6	6	6	6	6	6
6	6	6	6	6	6	6	6	6	6
6	6	6	6	6	6	6	6	6	6
6	6	6	6	6	6	6	6	6	6
4	4	4	4	4	4	4	4	4	4
5	5	6	6	6	6	6	6	6	6
4	4	4	3	3	3	4	4	4	4
5	5	5	4	5	4	4	4	5	4
6	6	3	6	4	6	5	4	5	6
6	6	5	6	5	6	5	6	5	5
6	5	5	6	5	6	5	5	5	6
6	5	4	5	4	5	5	4	5	5
4	4	3	5	4	3	4	4	5	4
5	5	5	4	5	4	4	3	5	4
4	4	3	5	4	3	4	4	5	4
5	5	5	5	5	5	5	5	5	5
4	4	5	5	4	6	4	5	5	5
4	4	5	5	4	6	4	5	5	5
3	4	4	4	3	2	2	1	3	1
5	4	5	5	5	5	5	5	4	5
5	4	5	4	4	5	4	5	4	5
4	4	4	4	5	5	4	5	5	4
6	6	6	6	6	6	5	5	5	6
5	4	5	5	5	5	5	5	5	5
5	5	4	5	5	4	4	5	5	5
4	4	4	4	4	4	4	4	4	4
4	5	5	5	5	5	5	5	5	5
5	4	5	5	5	5	5	5	5	5
4	5	5	5	5	5	5	5	5	5
5	5	5	5	5	5	5	5	5	5

5	5	5	5	5	5	5	5	5	5
3	4	4	4	3	2	2	1	3	1
5	5	5	4	4	5	4	5	4	5
5	6	5	5	5	5	5	5	5	4
4	4	4	4	3	4	2	4	2	4
4	4	3	4	3	6	4	4	3	4
6	4	4	3	3	3	5	4	4	5
4	5	5	4	3	4	5	5	5	5
5	5	5	5	5	5	5	5	5	5
6	6	3	4	2	6	6	4	6	6
5	5	2	5	1	6	5	1	2	5
5	5	5	5	5	5	5	5	5	5
4	5	4	3	4	5	5	5	4	3
2	3	2	1	2	2	3	2	2	3
5	5	4	5	5	4	5	6	5	6
5	4	5	4	4	5	6	5	4	5
4	3	3	4	3	5	5	4	5	4
5	4	3	4	5	6	6	5	4	4
6	5	4	6	4	6	4	6	4	5
3	3	4	4	4	3	3	3	5	4
4	4	4	4	4	4	5	4	4	4
6	6	4	5	3	6	5	6	5	6
5	5	4	4	2	5	4	5	3	5
5	5	4	3	3	4	4	3	5	5
2	3	2	3	2	3	3	2	2	3
5	4	3	4	4	3	4	5	4	4
6	6	3	5	2	5	5	6	5	5
6	4	6	6	4	6	6	6	6	6
5	5	4	5	4	5	5	6	5	5
4	5	2	5	2	4	4	6	4	5
5	5	4	5	4	5	5	6	5	5
5	5	3	5	1	5	6	6	6	6
6	6	3	5	3	6	5	6	5	6
5	5	4	5	4	5	5	5	5	5
6	6	4	5	3	6	5	6	5	6
5	5	4	4	2	5	4	5	3	5
3	3	4	4	4	4	4	4	4	4
3	4	4	4	4	4	3	4	3	4
4	3	4	4	3	4	4	4	4	4
6	6	5	5	4	5	6	4	5	6
6	4	5	5	6	6	6	5	3	6
5	5	4	4	4	4	5	5	4	4
3	4	4	4	4	4	4	4	4	4
5	5	6	6	6	6	5	6	5	6
4	4	4	5	5	4	4	4	5	5
5	5	5	4	4	4	4	5	5	4
3	4	4	4	4	4	4	4	4	4
5	5	5	5	5	5	5	5	5	5
4	5	4	5	4	5	4	5	4	5
5	5	5	5	5	5	5	5	5	5

5	6	6	5	5	5	5	6	5	6
3	2	3	3	3	3	5	4	5	3
5	4	4	6	6	5	6	4	5	4
6	6	5	1	3	2	2	4	3	5
4	4	6	4	6	4	5	6	5	4
4	4	4	5	3	4	4	5	4	5
5	5	5	5	5	5	5	5	5	5
6	6	6	6	6	5	5	5	6	5
4	3	3	4	3	2	3	4	3	3
6	6	6	4	6	6	5	6	5	6
4	4	5	6	5	6	5	6	5	5
4	4	4	4	4	4	4	5	5	5
6	6	5	5	4	6	6	6	6	6
3	4	5	4	3	4	4	5	4	4
4	4	4	4	4	4	4	4	4	4
5	5	5	6	5	4	4	5	6	5
4	4	5	4	4	5	4	4	5	4
5	5	5	6	5	5	6	6	5	5
4	4	5	3	4	5	6	6	4	6
3	3	3	4	5	5	4	4	3	4
5	5	5	4	5	4	4	5	5	4
4	4	4	4	5	5	5	4	4	4
4	4	5	4	4	3	4	4	4	4
3	4	4	3	4	4	4	4	4	4
5	4	5	3	4	5	4	3	4	5
4	4	4	5	4	5	4	4	4	4
3	4	3	2	1	3	4	3	3	5
4	4	4	4	4	4	4	4	4	4
4	4	3	4	4	5	4	5	3	4
6	5	6	5	6	5	4	5	6	5
3	3	3	3	3	3	3	3	3	3
4	3	4	3	4	3	4	3	4	3
5	4	5	5	4	4	5	4	5	4
4	5	2	4	2	4	5	3	4	4
4	4	5	5	5	4	4	5	5	6
4	5	4	4	4	5	6	5	4	6
5	5	5	5	5	5	5	5	5	5
4	4	4	4	5	5	5	4	4	4
5	5	5	5	5	5	5	5	5	5
4	3	4	3	5	3	4	4	4	4
6	6	5	6	5	6	5	6	4	6
4	4	5	4	5	4	6	5	6	4
5	3	5	5	4	4	4	5	4	4
5	5	5	5	5	6	6	6	6	5
6	5	4	4	4	5	6	6	6	6
6	6	4	5	6	6	4	5	5	4
4	3	2	3	2	4	5	6	5	6
4	3	4	5	5	4	5	6	6	5
3	4	3	4	4	4	5	6	5	4
4	4	3	4	4	3	4	4	4	4

4	5	4	4	5	4	5	4	6	5
3	4	5	5	4	3	4	3	3	2
4	5	5	5	5	5	5	5	5	5
3	4	4	3	2	3	5	4	3	2
4	4	4	4	4	4	3	4	3	3
5	5	4	5	6	5	5	6	4	5
3	4	4	3	4	3	4	3	3	4
5	5	5	4	4	5	5	4	4	4
1	1	2	2	1	4	1	1	1	1
1	1	1	1	1	1	1	1	1	1
4	4	4	4	4	4	4	4	4	4
6	6	6	6	6	6	6	6	6	6
6	4	4	5	5	4	4	5	4	5
6	6	6	6	6	6	6	6	6	6
4	4	2	3	2	4	4	4	4	4
6	6	6	5	5	6	6	6	6	6
4	4	4	4	3	4	5	5	6	4
6	5	4	5	5	6	6	5	4	6
6	6	6	6	6	6	6	6	6	6
6	6	5	5	4	6	5	5	5	6

PNS18
4
4
4
6
6
4
4
4
3
4
2
3
3
5
4
4
6
6
4
4
4
6
5
4
5
6
6
5
6
5
5
3
4
4
4
6
2
6
5
4
4
2
5
6
6
6
3
3
6

5
6
6
3
4
4
5
6
4
3
5
5
5
1
4
4
2
4
6
5
6
5
5
4
5
5
4
2
5
4
5
3
6
5
5
1
4
1
6
3
4
5
4
6
4
4
4
4
4
5

3
4
5
4
6
5
6
6
5
6
5
6
3
5
6
6
3
5
3
5
5
5
5
6
5
5
3
6
5
3
4
4
6
4
6
4
5
5
3
6
4
4
6
4
3
5
5
3
3
3

4
5
2
6
4
4
6
6
1
6
4
5
6
5
3
4
4
4
2
1
5
5
5
4
6
4
5
4
6
6
6
4
3
3
6
3
4
1
5
3
6
5
3
6
4
5
3
4
5
3

5
5
4
4
3
3
5
4
3
4
4
2
4
4
5
6
4
5
6
6
6
4
6
6
5
6
4
5
5
6
3
3
3
2
5
3
4
4
4
5
3
5
4
3
3
4
4
4
5
5

5
4
6
5
6
4
6
6
5
5
3
4
4
3
4
6
4
5
3
5
6
5
3
4
4
4
6
5
6
6
5
3
3
5
4
4
4
4
3
5
3
4
3
4
3
6
6
5
5
5

5
5
5
4
5
5
5
5
6
2
6
5
6
6
5
4
5
5
6
6
5
6
4
5
3
3
5
3
5
6
4
4
4
6
3
5
4
4
4
3
4
4
4
3
6
4
4
6
5
6

5
3
6
4
4
3
5
2
4
6
6
5
5
4
3
6
6
5
5
6
4
5
3
5
4
4
5
5
4
3
4
4
5
6
4
5
5
5
3
6
5
6
5
6
6
6
6
5
6
5

4
6
6
5
5
3
6
5
5
5
5
6
6
4
4
3
4
4
3
4
5
5
6
2
5
5
5
5
4
3
6
6
5
5
5
5
5
3
6
6
6
4
6
4
5
2
5
5
5
4

6
5
6
4
6
6
6
6
5
4
6
3
3
2
6
5
6
4
3
2
4
2
6
2
6
4
5
4
5
4
5
4
6
4
6
4
6
3
5
6
6
4
5
6
5
5
5
4
5
3

4
6
4
6
5
6
2
3
6
5
2
6
6
4
3
6
5
6
4
6
6
6
3
4
5
5
6
3
6
4
6
4
6
5
4
6
6
4
3
4
5
6
6
6
5
6
6
6
6
5

4
4
6
4
6
5
6
6
6
4
5
3
6
5
6
4
4
5
3
3
3
6
3
6
6
6
5
4
4
6
4
5
4
4
5
4
5
5
4
2
5
4
4
4
3
4
6
4
3
4

3
3
6
4
5
3
5
6
6
4
5
6
4
4
5
6
3
6
3
6
5
4
5
5
4
4
5
5
5
5
6
4
4
4
4
4
5
5
2
6
5
6
4
5
6
6
5
3
4
5

3
5
3
3
4
3
5
5
4
5
5
5
4
4
5
6
4
5
4
5
6
6
4
5
6
5
3
4
4
6
5
3
4
5
4
4
3
3
4
6
5
4
5
3
6
3
4
3
3
4

6
3
3
4
5
5
6
4
4
2
6
3
5
4
4
4
3
4
5
4
5
5
3
3
2
2
2
4
4
4
5
5
5
2
4
6
5
4
4
4
6
6
4
4
4
5
5
6
6
6

3
3
6
3
6
4
4
4
3
4
5
6
4
6
5
6
4
4
5
4
6
6
6
6
4
5
4
5
6
6
6
5
5
5
5
5
5
5
3
5
5
5
6
5
5
4
5
5
5
5

5
3
5
4
4
6
3
4
6
6
6
5
3
2
6
6
4
6
4
4
4
6
6
5
2
4
5
6
5
5
5
6
6
5
6
6
4
4
4
5
6
4
4
6
5
4
3
5
5
5

5
2
3
5
4
4
5
5
4
6
5
4
6
4
4
4
5
5
3
4
4
4
4
4
5
4
6
4
4
4
3
4
4
4
5
3
5
5
5
3
6
5
4
6
6
4
6
4
5
5

5
3
5
2
4
6
4
5
1
1
4
6
4
6
4
6
6
6
6
6
